# Identifying gut microbiota *Faecalibacterium* as a potential biomarker for distinguishing visceral or subcutaneous obese population

**DOI:** 10.3389/fmicb.2025.1635962

**Published:** 2025-08-29

**Authors:** Yi Li, Senlin Wang, Song Zhang, Ergan Li, Meifang Liang, Youqin Li, Anke Liuli, Li Deng, Yanjun Liu, Tongtong Zhang, Di Xin, Yongmei Li, Zhonghui Feng

**Affiliations:** ^1^Department of Radiology, The First Affiliated Hospital of Chongqing Medical University, Chongqing, China; ^2^Department of Radiology, The Third People’s Hospital of Chengdu, Chengdu, Sichuan, China; ^3^Division of Liver Surgery, Department of General Surgery, West China Hospital, Sichuan University, Chengdu, China; ^4^Obesity and Metabolism Medicine-Engineering Integration Laboratory, Department of General Surgery, The Affiliated Hospital of Southwest Jiaotong University, The Third People’s Hospital of Chengdu, Chengdu, China; ^5^The Center of Obesity and Metabolic Diseases, Department of General Surgery, The Affiliated Hospital of Southwest Jiaotong University, The Third People’s Hospital of Chengdu, Chengdu, Sichuan, China; ^6^College of Medicine, Southwest Jiaotong University, Chengdu, China; ^7^Medical Research Center, The Affiliated Hospital of Southwest Jiaotong University, The Third People’s Hospital of Chengdu, Chengdu, China; ^8^Department of Pediatrics, Affiliated Hospital of Qingdao University, Qingdao, Shandong, China; ^9^College of Animal Science and Veterinary Medicine, Southwest Minzu University, Chengdu, China; ^10^College of Life Science and Engineering, Southwest Jiaotong University, Chengdu, China; ^11^Section for Hepato Pancreato Biliary Surgery, Department of General Surgery, Affiliated Hospital of Southwest Jiaotong University, The Third People’s Hospital of Chengdu, Chengdu, China; ^12^Research Center for Obesity and Metabolic Health, College of Medicine, Southwest Jiaotong University, The Third People’s Hospital of Chengdu, Chengdu, China; ^13^Department of Hepatobiliary and Pancreatic Surgery and Zhejiang Provincial Key Laboratory of Pancreatic Disease, The First Affiliated Hospital, Zhejiang University School of Medicine, Hangzhou, Zhejiang, China

**Keywords:** obesity, visceral fat, subcutaneous fat, gut microbiota, *Faecalibacterium*

## Abstract

**Background:**

Obesity is a global health issue, with sharply increasing rates due to excessive food intake and reduced physical activity, leading to an increased risk of various chronic diseases, such as type 2 diabetes and cardiovascular diseases. Fat distribution plays a significant role in health, with visceral fat being particularly associated with metabolic syndrome. Currently, computed tomography (CT) and magnetic resonance imaging (MRI) are the gold standards for measuring visceral fat, but they are costly and involve radiation risks. The gut microbiota is closely related to obesity, and dysbiosis may lead to obesity and metabolic disorders. Research on the relationship between visceral fat and the gut microbiota can aid in the development of new diagnostic and therapeutic approaches.

**Methods:**

We selected 31 participants with class II obesity (body mass index between 35 and 40) and divided those samples into two groups on the basis of their VSR (visceral-to-subcutaneous fat volume ratio) measured by CT, analyzed their fecal. Microbiota through 16S rDNA sequencing.

**Results:**

Results reveal significant differences in microbial composition between the visceral and subcutaneous obesity groups through 16S analysis of their fecal microbiota. The visceral obesity group presented a greater abundance of the *Blautia* genus, whereas the subcutaneous obesity group presented a greater abundance of the *Faecalibacterium* genus. Species difference analysis and clinical correlation analysis revealed that *Blautia* and *Faecalibacterium* were associated with visceral and subcutaneous obesity, respectively, and played opposite roles. Moreover, in our validation cohort (*n* = 16), we also found that the subcutaneous obesity group had a greater abundance of the *Faecalibacterium* genus.

**Conclusion:**

This study measured visceral and subcutaneous fat volumes via CT and revealed that the composition of the gut microbiota is related to the type of obesity. In addition, we found that *Blautia* and *Faecalibacterium* were associated with visceral and subcutaneous obesity, which provides new insights for the diagnosis and treatment of obesity.

## 1 Introduction

Obesity is a global public health concern characterized by abnormal or excessive accumulation of body fat, which poses a significant risk to health and well-being. Because of excessive food intake and decreased physical activity, Obesity has increased dramatically over recent decades and become a global health crisis (Hill and Peters, 1998; Sallis and Glanz, 2009). Obesity is a key risk factor for chronic conditions like type 2 diabetes, cardiovascular diseases, fatty liver disease, musculoskeletal disorders, and certain cancers, which can significantly reduce the quality of life and life expectancy (Piche et al., 2020; Polyzos et al., 2019; Lin and Li, 2021).

Fat distribution, or where the body stores fat, plays a critical role in health outcomes and is an important factor in assessing disease risk beyond just considering total body fat or weight (Li and Qi, 2019). Different patterns of fat distribution are associated with varying levels of risk for metabolic and cardiovascular diseases. Fat stored in the abdominal area, particularly visceral fat, is more metabolically active and has been linked to a higher risk of metabolic syndrome, which includes high blood pressure, high blood sugar, abnormal cholesterol levels, and excess abdominal fat (Neeland et al., 2019; Ibrahim, 2010). Visceral fat and subcutaneous fat are two distinct types of adipose tissue with different health implications. Visceral fat surrounds internal organs and is more metabolically active, increasing the risk of metabolic diseases like type 2 diabetes and cardiovascular conditions. Subcutaneous fat, located under the skin, is generally less harmful.

The measurement of visceral fat is crucial for assessing obesity-related health risks. Currently, computed tomography (CT) and magnetic resonance imaging (MRI) are considered the gold standards in quantifying visceral fat due to their high accuracy. However, these methods are costly, less accessible, and involve exposure to radiation or magnetic fields. Alternatively, dual-energy X-ray absorptiometry (DXA) and ultrasound can also estimate visceral fat but with lower precision. Anthropometric measures like waist circumference and waist-to-hip ratio serve as indirect indicators of visceral fat but lack specificity (Fox et al., 2007; Smith et al., 2001). The development of a more affordable, simpler, and safer method for measuring and assessing visceral fat would be a significant advancement in public health.

Gut microbiota, the collection of microorganisms in the digestive tract, has a significant role in regulating various physiological processes, including metabolism, energy balance, and immune function. The relationship between gut microbiota and obesity has garnered attention in recent research, indicating that imbalances in these microbial communities, termed dysbiosis, can contribute to obesity and related metabolic disorders. The gut microbiota, also known as the gut flora, refers to the diverse community of microorganisms that inhabit the gastrointestinal tract of humans and other animals. This complex ecosystem plays a crucial role in health and disease, influencing various aspects of host physiology, including digestion, immunity, and metabolism (Sender et al., 2016; Turnbaugh et al., 2006; Ley et al., 2006; Ridaura et al., 2013).

The gut microbiota plays a critical role in the regulation of body fat distribution. An altered gut microbiota, known as dysbiosis, has been associated with increased visceral adiposity and its related metabolic consequences (Vrieze et al., 2012; Romani-Perez et al., 2024; Baothman et al., 2016; Valsecchi et al., 2016). Studies suggest that gut microbiota influences fat storage and energy regulation through several mechanisms containing modification of energy extraction from the diet, microbial metabolites such as short-chain fatty acids (SCFAs), and systemic inflammation (Valsecchi et al., 2016; Harakeh et al., 2016). Understanding the relationship between visceral fat and gut microbiota is critical for developing novel diagnostic and therapeutic strategies for obesity and its related disorders.

Recent studies have begun to elucidate the intricate relationship between visceral fat and gut microbiota (Yan et al., 2021). However, they chose people with normal body mass index (BMI) (18.5 ≤ BMI < 23.9) and divided the people into the low visceral fat area (L-VFA) group (VFA < 100 cm^2^) and the high visceral fat area (H-VFA) group (VFA ≥ 100 cm^2^) by visceral fat area. Studies show that visceral fat volume, compared to area, more closely represents total body fat distribution (Fang et al., 2018; Ozer Cakir and Yildiz, 2016), and measurements of fat distribution at the L2–L3 position better reflect total body fat distribution (Cheng et al., 2018). VAT is an important risk factor for metabolic diseases, while SAT may have a relatively protective role (Bunnell et al., 2021), so recent studies have used the VSR (the visceral-to-subcutaneous fat ratio) to assess visceral obesity (Kim et al., 2022).

We selected 31 participants in the discovery cohort with class II obesity (35 ≤ BMI < 40) and divided them into two groups based on their VSR, discovering significant differences in microbial composition between the visceral and subcutaneous obesity groups through 16S analysis of their fecal microbiota. The visceral obesity group showed a higher abundance of the *Blautia* genus, while the subcutaneous obesity group had a higher abundance of the *Faecalibacterium* genus. Species difference analysis and clinical correlation analysis revealed that *Blautia* and *Faecalibacterium* were associated with visceral and subcutaneous obesity, respectively, and played opposite roles. Moreover, in our validation cohort (*n* = 16), we also found that the subcutaneous obesity group had a higher abundance of the *Faecalibacterium* genus.

This study could pave the way for novel diagnostic and therapeutic approaches for obesity and its related conditions. Gut microbiota profiling in individuals with high visceral fat may reveal specific microbial signatures that contribute to or result from visceral fat accumulation. These microbial markers could serve as diagnostic tools and non-invasive biomarkers to identify individuals at risk of visceral obesity and its associated complications.

## 2 Materials and methods

### 2.1 Study population

This study is divided into a discovery cohort (*n* = 31) and a validation cohort (*n* = 16). The detailed flowchart can be found in the Supplementary Figure 1. To ensure good comparability between the two groups of participants, we strictly limited the BMI range to individuals with a BMI between 35 and 40 (35 ≤ BMI < 40). In the validation cohort, the BMI range was extended to 28–50 (28 ≤ BMI < 50) to broaden the applicability of the study and further validate our findings. Upper abdominal scanning was performed on participants using a 64-slice spiral CT or dual-source CT. Subsequently, the visceral fat volume (VFV) and subcutaneous fat volume (SFV) within the region of the third lumbar vertebra (L3) were measured using the open-source software Syngo. In both the discovery and validation cohorts, subjects were grouped based on the visceral-to-subcutaneous fat ratio (VSR, defined as the ratio of VFV to SFV). When VSR < 0.5, participants were classified as the control group (subcutaneous obesity); when VSR > 0.9, they were classified as the experimental group (visceral obesity). All studies involving human participants were reviewed and approved by the Ethics Committee of the Third People’s Hospital of Chengdu (Approval No.: 2022-S-62), with each participant signing an informed consent form prior to enrollment.

### 2.2 Study participants and inclusion/exclusion criteria

This study employed stringent inclusion and exclusion criteria to ensure participant homogeneity. The inclusion criteria were as follows: (Hill and Peters, 1998) age 18–65 years; (Sallis and Glanz, 2009) BMI ≥ 28 kg/m^2^ (based on Chinese obesity criteria); (Piche et al., 2020) confirmed diagnosis of visceral or subcutaneous obesity via CT; and (Polyzos et al., 2019) no antibiotic use within the preceding 2 months (Palleja et al., 2018). Exclusion criteria comprised: (Hill and Peters, 1998) current or prior history of infectious diseases, including viral hepatitis, tuberculosis, or typhoid fever; (Sallis and Glanz, 2009) use of immunosuppressive agents within the past 3 months; (Piche et al., 2020) concurrent inflammatory bowel disease; and (Polyzos et al., 2019) pregnancy or lactation. Prior to enrollment, all participants underwent comprehensive screening, including complete blood count, liver and kidney function tests, and infectious disease screening.

### 2.3 Basic information and biochemical indicators

Basic clinical information was collected from participants, including age, triglycerides, blood glucose, high-density lipoprotein (HDL), cholesterol, fasting blood glucose (FBG), and glycated hemoglobin (HbA1C). Body weight was measured using an SMF-BIA device (Inbody770, Biospace, South Korea) with participants dressed in light clothing, and readings were recorded to the nearest 0.1 kg. Height was measured with a stadiometer accurate to 0.1 cm. BMI was calculated by dividing weight (kg) by height squared (m^2^). Blood biochemical markers were obtained via venipuncture after participants had fasted, using sterile needles. The samples were processed and stored based on the test requirements, kept under low-temperature conditions, and delivered to the laboratory within the specified time for analysis using a Roche chemical luminescence analyzer (Germany).

### 2.4 CT scan and analysis

**Figure 1:**
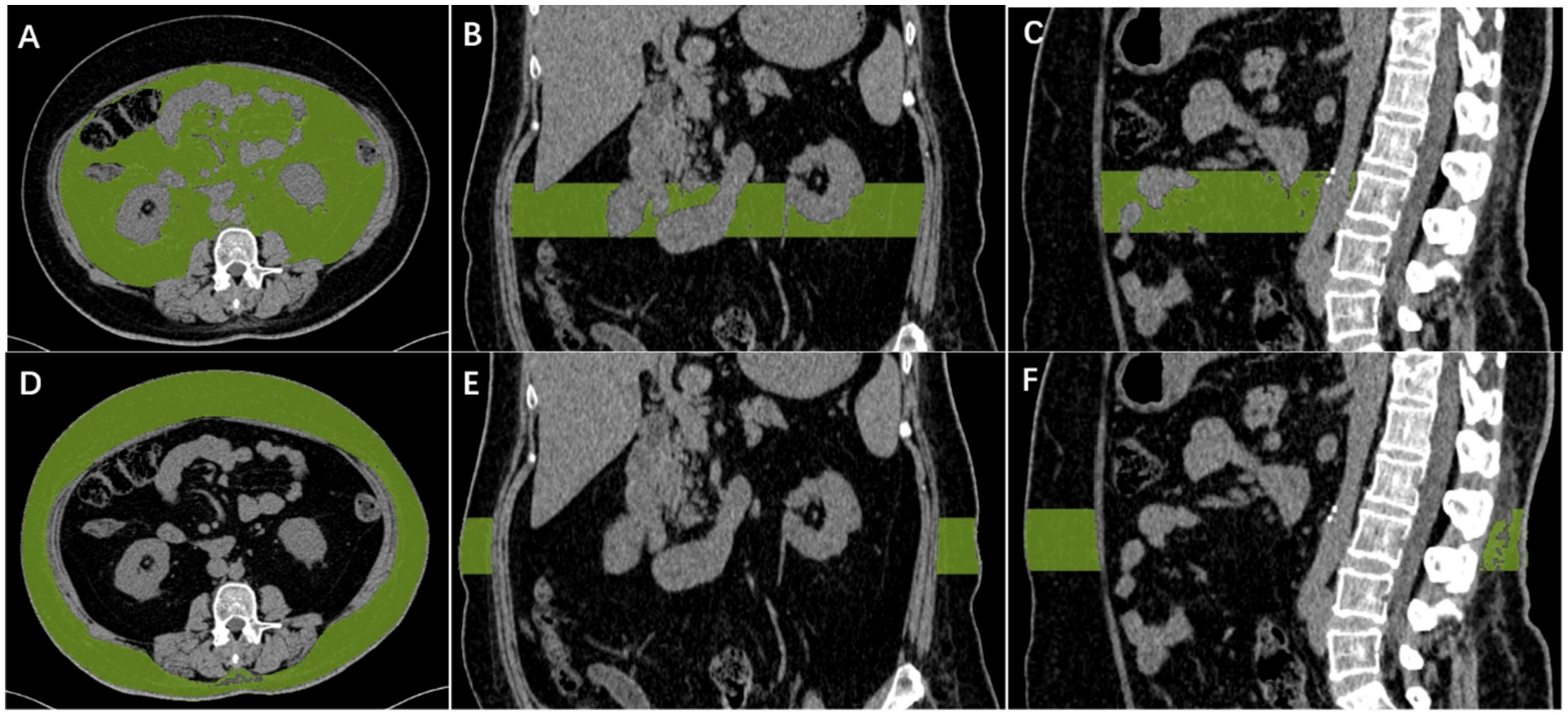
Diagram of measurement of visceral fat and subcutaneous fat. Automatic calculation of visceral fat volume in the L3 vertebral range, with CT density between −190 and −30 HU for visceral fat **(A–C)**. Automatic calculation of subcutaneous fat volume in the L3 vertebral range, with CT density between −190 and −30 HU for subcutaneous fat **(D–F)**.

CT of the abdomen was performed on a 64-slice multidetector CT or dual-source CT scanner (Somatom Definition AS, Siemens Healthineers, Germany or SOMATOM Force, Siemens Healthineers, Germany), tube voltage, 120 kV; tube current variable mAs (maximum 400 mAs); pitch, 0.5; collimation, 0.6 mm; reconstructed slice thickness, 1.5 mm. The axial CT images containing L3 vertebral bodies were exported and further processed with the open-source software Syngo. Via. On the L3 vertebral articular surface level, manually Outline the scope of visceral fat, use the mouse wheel will level switch to L3 vertebral articular surface level, continue to manually sketch the outline of visceral fat, and then click the “insert outline” and then “add region,” then apply fat specificity thresholds (− to −30 HU, 190) (Shen et al., 2022; Kim et al., 2021; Lee et al., 2021). Visceral fat volume was measured throughout the L3 vertebral body. The same method was used to derive the subcutaneous fat volume. For details on performing a detailed CT scan, please refer to the Figure 1.

### 2.5 DNA extraction and PCR amplification

Total microbial genomic DNA was extracted from samples using the PF Mag-Bind Stool DNA Kit (Omega Bio-tek, Georgia, U.S.) according to the manufacturer’s instructions. The quality and concentration of DNA were determined by 1.0% agarose gel electrophoresis and a NanoDrop® ND-2000 spectrophotometer (Thermo Scientific Inc., USA) and kept at −80°C prior to further use. The hypervariable region V3-V4 of the bacterial 16S rRNA gene was amplified with primer pairs 338F (5′-ACTCCTACGGGAGGCAGCAG-3′) and 806R (5′-GGACTACHVGGGTWTCTAAT-3′) (Liu et al., 2016) by an ABI GeneAmp® 9,700 PCR thermocycler (ABI, CA, USA). The PCR reaction mixture including 4 μL 5 × Fast Pfu buffer, 2 μL 2.5 mM dNTPs, 0.8 μL each primer (5 μM), 0.4 μL Fast Pfu polymerase, 0.2 μL BSA, 10 ng of template DNA, and ddH_2_O to a final volume of 20 μL. PCR amplification cycling conditions were as follows: initial denaturation at 95°C for 3 min, followed by 27 cycles of denaturing at 95°C for 30s, annealing at 55°C for 30s, and extension at 72°C for 45s, and single extension at 72°C for 10 min, and end at 4°C. All samples were amplified in triplicate. The PCR product was extracted from 2% agarose gel and purified. Then quantified using Quantus™ Fluorometer (Promega, USA).

### 2.6 Amplicon sequence processing and analysis

After demultiplexing, the resulting sequences were quality-filtered with fastp (0.19.6) (Magoc and Salzberg, 2011) and merged with FLASH (v1.2.11). Then the high-quality sequences were de-noised using DADA2 (Callahan et al., 2016) plugin in the Qiime2 (Amir et al., 2017) (version 2020.2) pipeline with recommended parameters, which obtains single nucleotide resolution based on error profiles within samples. DADA2-denoised sequences are usually called amplicon sequence variants (ASVs). To minimize the effects of sequencing depth on alpha and beta diversity measure, the number of sequences from each sample was rarefied to 20,000, which still yielded an average Good coverage of 99.90%. Taxonomic assignment of ASVs was performed using the Naive Bayes consensus taxonomy classifier implemented in Qiime2 and the SILVA 16S rRNA database (v138).

### 2.7 DNA extraction and qPCR

The total genomic DNA of microbial communities was extracted from visceral obesity and subcutaneous obesity fecal samples according to the instructions of the FastPure Stool DNA Isolation Kit (MJYH, Shanghai, China). The integrity of the extracted genomic DNA was examined by agarose gel electrophoresis with 1% agarose, and the concentration and purity of the DNA were determined by using a NanoDrop2000 (Thermo Fisher Scientific, USA). The concentration and purity of the DNA were determined using a NanoDrop2000 instrument (Thermo Scientific, USA). The validation of qPCR was then conducted in accordance with the instructions provided by the manufacturer of Taq Pro Universal SYBR qPCR Master Mix (Vazyme Biotech Co., Ltd., China). The resulting data were subsequently calculated using the 2^−ΔΔCt^ method. The number of samples was visceral obesity (*n* = 6) and subcutaneous obesity (*n* = 6). qPCR primers is listed in Supplementary Table 2.

### 2.8 Statistical analysis

Bioinformatic analysis of the gut microbiota was carried out using the Majorbio Cloud platform.^1^ Based on the ASVs information, rarefaction curves and alpha diversity indices including observed ASVs, Chao1 richness, Shannon index, and Good’s coverage were calculated with Mothur v1.30.2 (Barberan et al., 2012). The similarity among the microbial communities in different samples was determined by principal coordinate analysis (PCoA) based on Bray–Curtis dissimilarity using the Vegan v2.4.3 package. The PERMANOVA test was used to assess the percentage of variation explained by the treatment along with its statistical significance using the Vegan v2.4.3 package. The linear discriminant analysis (LDA) effect size (LEfSe) (Douglas et al., 2020)^2^ was performed to identify the significantly abundant taxa (phylum to genera) of bacteria among the different groups (LDA score > 2, *p* < 0.05). The distance-based redundancy analysis (db-RDA) was performed using the Vegan v2.4.3 package to investigate the effect of clinical parameters on gut bacterial community structure. Forward selection was based on Monte Carlo permutation tests (permutations = 9,999). Values of the x- and y-axes and the length of the corresponding arrows represented the importance of each clinical parameter in explaining the distribution of taxons across communities. Linear regression analysis was applied to determine the association between major clinical parameters identified by db-RDA analysis and microbial alpha diversity indices. The analysis of the correlation between clinical parameters and microbial community species was conducted using the MaAsLin2 package. The co-occurrence networks were constructed to explore the internal community relationships across the samples (Bolyen et al., 2019). The metagenomic function was predicted by PICRUSt2 (Phylogenetic Investigation of Communities by Reconstruction of Unobserved States) (Segata et al., 2011) based on ASV representative sequences. PICRUSt2 is a software containing a series of tools as follows: HMMER was used to align ASV representative sequences with reference sequences. EPA-NG and Gappa were used to put ASV representative sequences into a reference tree. The castor was used to normalize the 16S gene copies. MinPath was used to predict gene family profiles and locate the gene pathways. The entire analysis process was according to the protocols of PICRUSt2. A correlation between two nodes was considered to be statistically robust if Spearman’s correlation coefficient was over 0.6 or less than −0.6, and the *p*-value < 0.05.

## 3 Results

### 3.1 Clinical characteristics of subjects

The baseline characteristics of participants in the discovery cohort revealed significant differences between the visceral obesity and subcutaneous obesity groups (Table 1). Visceral obesity was associated with a significantly higher VFV and VSR, while the SFV was greater in the subcutaneous obesity group. Other clinical parameters, including BMI, HbA1c, and various metabolic markers, did not show significant differences between the groups. Similar characteristics were also observed in the validation cohort (Supplementary Table 1).

**Table 1:** Baseline characteristics of participants in the discovery cohort.

Characteristics	Visceral obesity (*n* = 17)	Subcutaneous obesity (*n* = 14)	*p*-values
VFV	1155.58 (240.86)	606.89 (155.85)	<0.001
SFV	1075.70 (235.67)	1594.97 (296.83)	<0.001
VSR	1.13 (0.36)	0.39 (0.13)	<0.001
Gender, male, %	9 (52.94)	6 (42.86)	0.843
Age	37.88 (7.70)	33.50 (8.08)	0.134
BMI	36.52 (1.50)	37.43 (1.34)	0.088
HbAlc%	6.62 (1.40)	6.51 (1.60)	0.841
Folic acid	8.44 (3.55)	7.70 (2.72)	0.529
Albumin	44.28 (2.68)	42.46 (3.26)	0.098
Total protein	72.75 (5.20)	72.18 (3.98)	0.737
GGT	99.39 (58.25)	62.66 (62.48)	0.102
ALT	81.49 (66.29)	57.58 (33.67)	0.231
Calcium	2.34 (0.07)	2.31 (0.09)	0.29
AST	49.68 (46.46)	31.39 (13.87)	0.167
HDL-C	1.14 (0.18)	1.17 (0.19)	0.699
IBIL	10.24 (3.79)	9.89 (3.01)	0.783
TBIL	11.92 (3.75)	12.06 (3.45)	0.915
TG	2.84 (1.64)	2.78 (4.76)	0.963
LDL-C	3.03 (0.30)	3.14 (0.75)	0.584
Glucose	6.59 (3.78)	6.70 (3.16)	0.933
DBIL	2.30 (1.08)	2.17 (0.57)	0.677
Prealbumin	275.76 (49.46)	262.97 (71.49)	0.561
Total cholesterol	5.03 (0.60)	5.35 (1.25)	0.348
Iron	10.21 (3.38)	10.11 (2.59)	0.925
C-Peptide	1.76 (0.69)	1.44 (0.44)	0.143
Insulin	36.09 (20.85)	32.61 (21.14)	0.649
25(OH)D	36.75 (13.39)	30.68 (9.73)	0.168
Thyroxine	104.25 (24.67)	95.94 (18.42)	0.306
Hemoglobin	149.82 (15.67)	142.64 (16.79)	0.229

*p* < 0.05 was considered statistically significant. VFV, visceral fat area; SFV, subcutaneous fat area; VSR, visceral-to-subcutaneous fat ratio; HbAlc, hemoglobin A1c; GGT, gamma-glutamyl transferase; ALT, alanine aminotransferase; AST, aspartate aminotransferase; HDL-C, high-density lipoprotein cholesterol; IBIL, indirect bilirubin; TBIL, total bilirubin; TG, triglycerides; LDL-C, low-density lipoprotein cholesterol; DBIL, direct bilirubin; 25(OH)D, 25-hydroxy vitamin D; T4, Thyroxine.

### 3.2 Microbiota diversity analysis

**Figure 2:**
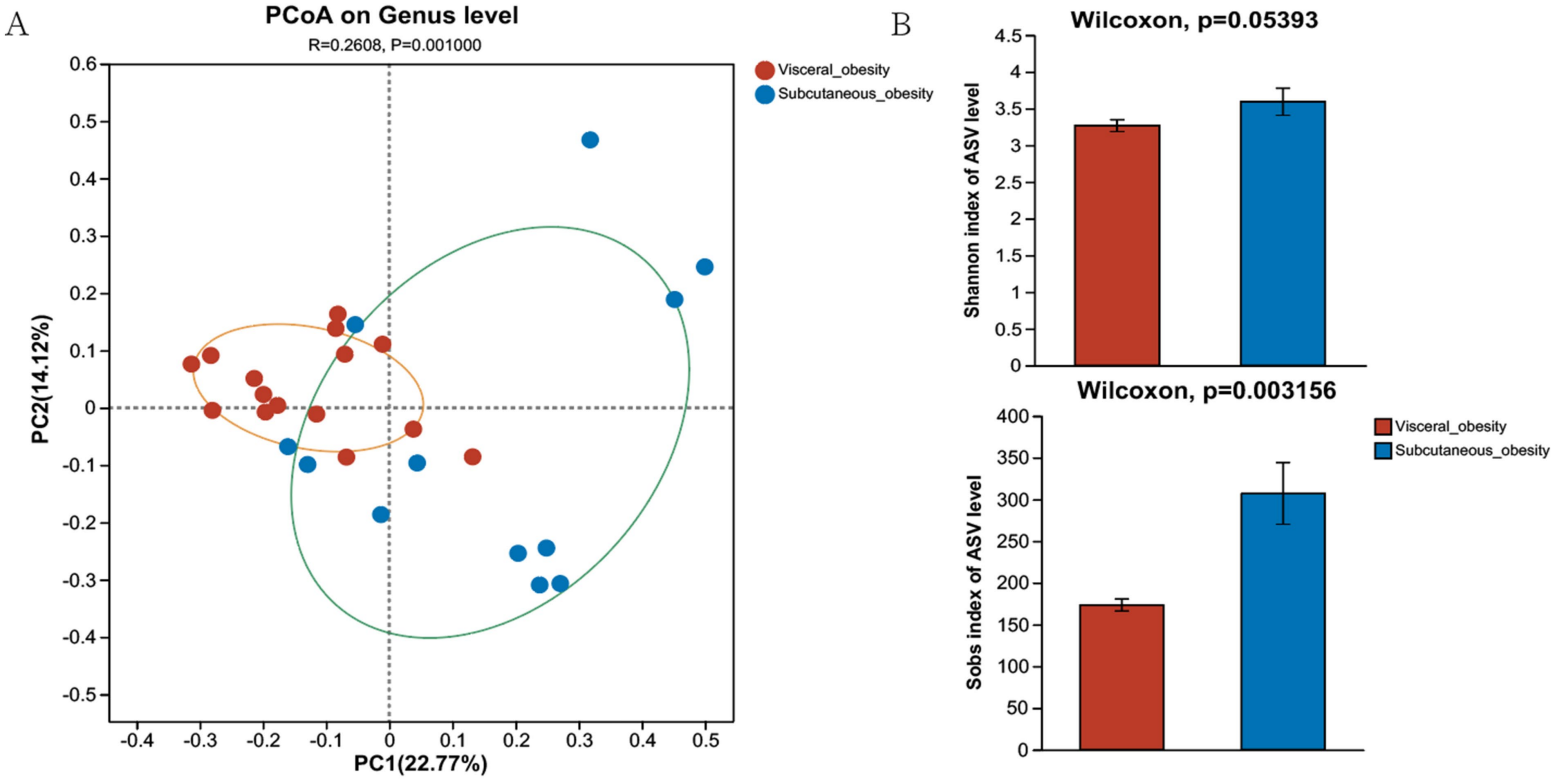
Composition and diversity of the microbiome in the discovery cohort. **(A)** Genus-level PCoA shows sample distribution for visceral and subcutaneous obesity, with confidence ellipses in orange and green. **(B)** Alpha diversity comparison (Shannon and Sob indices) shows higher diversity in the subcutaneous obesity group.

Figure 2A presents the PCoA at the genus level, showing a significant separation in the microbial community composition between the visceral obesity group and the subcutaneous obesity group (*R* = 0.2808, *p* = 0.001). This result was consistently validated using different distance metrics. As shown in Figure 2B, the Shannon and Sob diversity indices further support this separation. A similar pattern was observed at the family level (Supplementary Figure 2A; *R* = 0.2217, *p* = 0.004), with Bray–Curtis distance (Supplementary Figure 2B; *p* = 0.001345) and Hellinger distance (Supplementary Figure 2D; *p* = 0.0512) confirming the differences between the two obesity types. Wilcoxon tests indicated significant differences in microbial diversity indices between the groups, particularly for the Shannon index (Supplementary Figure 2C; *p* = 0.003692).

### 3.3 Analysis of the microbiota composition and associations between the gut microbiome and clinical characteristics

#### 3.3.1 Analysis of the microbiota composition

**Figure 3:**
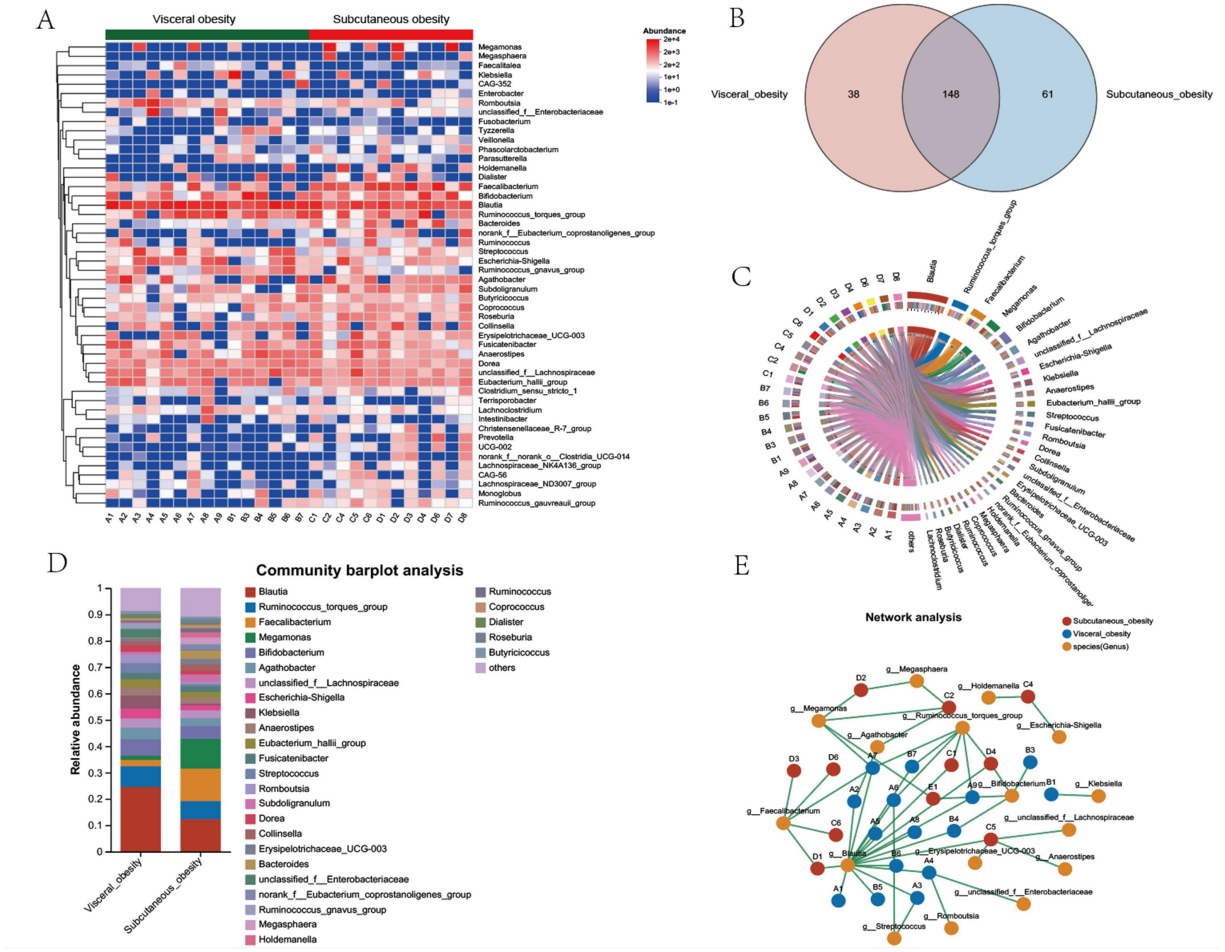
Microbial community composition, shared genera, and network analysis between visceral and subcutaneous obesity in the discovery cohort. **(A)** Heatmap showing the relative abundance of bacterial genera in visceral obesity and subcutaneous obesity groups. The color scale represents the abundance of each genus, with red indicating higher abundance and blue indicating lower abundance. **(B)** Venn diagram illustrating the number of shared and unique genera between visceral obesity (red) and subcutaneous obesity (blue) groups. A total of 148 genera are shared, while 38 are unique to visceral obesity and 61 are unique to subcutaneous obesity. **(C)** Circos plot depicting the relationships between the dominant bacterial genera and individual participants in visceral and subcutaneous obesity groups. The links highlight the bacterial genera that are more associated with either obesity type. **(D)** Community bar plot analysis showing the relative abundance of bacterial genera in visceral obesity and subcutaneous obesity groups. Different colors represent various genera, with the most abundant genera, such as *Blautia*, *Ruminococcus_torques_group*, and *Faecalibacterium* displayed for both groups. **(E)** Network analysis illustrating the interactions between bacterial genera (orange) and their associations with visceral obesity (green nodes) and subcutaneous obesity (red nodes). The lines connecting the nodes indicate co-occurrence or exclusion relationships among the bacterial genera, highlighting differences in microbial networks between the two obesity types.

The heatmap in Figure 3A provides a detailed comparison of the relative abundance of microbial taxa between the visceral and subcutaneous obesity groups. This analysis highlights key differences in microbial composition, with certain taxa showing significant changes. Notably, *Megamonas*, *Megasphaera*, and *Faecalibacterium* were more abundant in the subcutaneous obesity group, while *Blautia* and *Ruminococcus_gnavus_group* were more associated with subcutaneous visceral obesity. Figure 3B (Venn diagram) and Figure 3C (circular plot) further categorize the shared and unique genera between the two groups. It was found that 148 genera were shared between visceral and subcutaneous obesity, with 38 genera unique to visceral obesity and 61 unique to subcutaneous obesity. Figures 3D,E shows the distribution of the relative abundance of microbial genera in the visceral and subcutaneous obesity groups. The results indicate significant differences in microbial composition between the two groups, with the visceral obesity group showing a higher abundance of the genus *Blautia*, while the subcutaneous obesity group had higher levels of *Faecalibacterium*.

Supplementary Figure 3 presents a comparison of the microbial community composition between the visceral and subcutaneous obesity groups through phylogenetic trees and bar plot analyses. Supplementary Figure 3A displays a phylogenetic tree highlighting the phylogenetic distribution of microbial taxa that differ significantly between the visceral obesity (red) and subcutaneous obesity (blue) groups. The analysis reveals distinct microbial lineages enriched in each group. Notably, taxa within the phylum *Bacteroidetes*, such as *Bacteroides* and *Christensenellales*, were more abundant in the subcutaneous obesity group. Conversely, taxa within the phylum Firmicutes, such as *Lachnospiraceae* and *Erysipelatoclostridium*, were significantly enriched in the visceral obesity group. Supplementary Figure 3B shows a bar plot analysis at the order level, with *Lachnospirales* having higher relative abundance in the visceral obesity group, while *Oscillospirales* predominated in the subcutaneous obesity group. Supplementary Figure 3C provides a bar plot analysis at the family level, with *Lachnospiraceae* being significantly enriched in the visceral obesity group, while *Ruminococcaceae* was more abundant in the subcutaneous obesity group.

#### 3.3.2 Differential genera analysis in visceral and subcutaneous obesity

**Figure 4:**
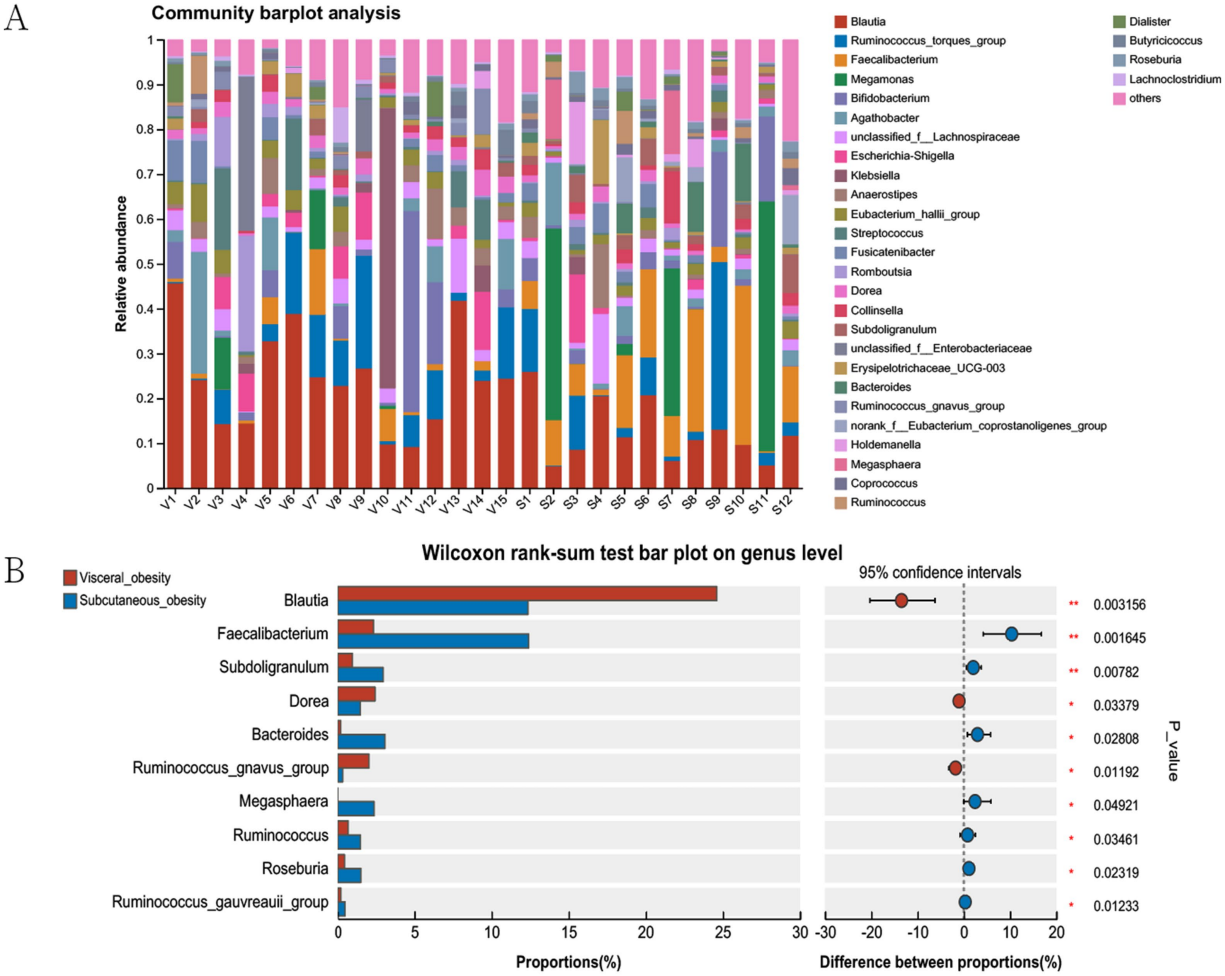
Microbial community analysis in visceral and subcutaneous obesity in the discovery cohort. **(A)** Community barplot showing the relative abundance of genera in visceral and subcutaneous obesity groups. **(B)** Wilcoxon rank-sum test comparing genus proportions between groups, with differences and statistical significance indicated. The bars represent the proportion (%) of each genus, with red and blue colors corresponding to visceral and subcutaneous obesity groups, respectively. The right side of the plot shows the difference in proportions between the groups, with confidence intervals and associated *p*-values indicating statistical significance.

Figure 4A shows bar plots of the relative abundance of microbial communities in different obesity groups (visceral obesity group V1–V15 and subcutaneous obesity group S1–S12). In the visceral obesity group, *Blautia* (red) and *Ruminococcus torques* group (blue) represented a significant proportion. In the subcutaneous obesity group, certain genera like *Megamonas* (green) and *Faecalibacterium* (orange) exhibited higher relative abundance. Figure 4B illustrates significant associations between genera in the two obesity groups, highlighting that *Blautia* is associated with the visceral obesity group, while *Faecalibacterium* is linked to the subcutaneous obesity group. Figure 4B indicates that genera such as *Blautia*, *Faecalibacterium*, and *Subdoligranulum* show statistically significant differences between the two groups (*Blautia*, *p* = 0.003156; *Faecalibacterium*, *p* = 0.016145).

#### 3.3.3 Associations between the gut microbiome and clinical characteristics

**Figure 5:**
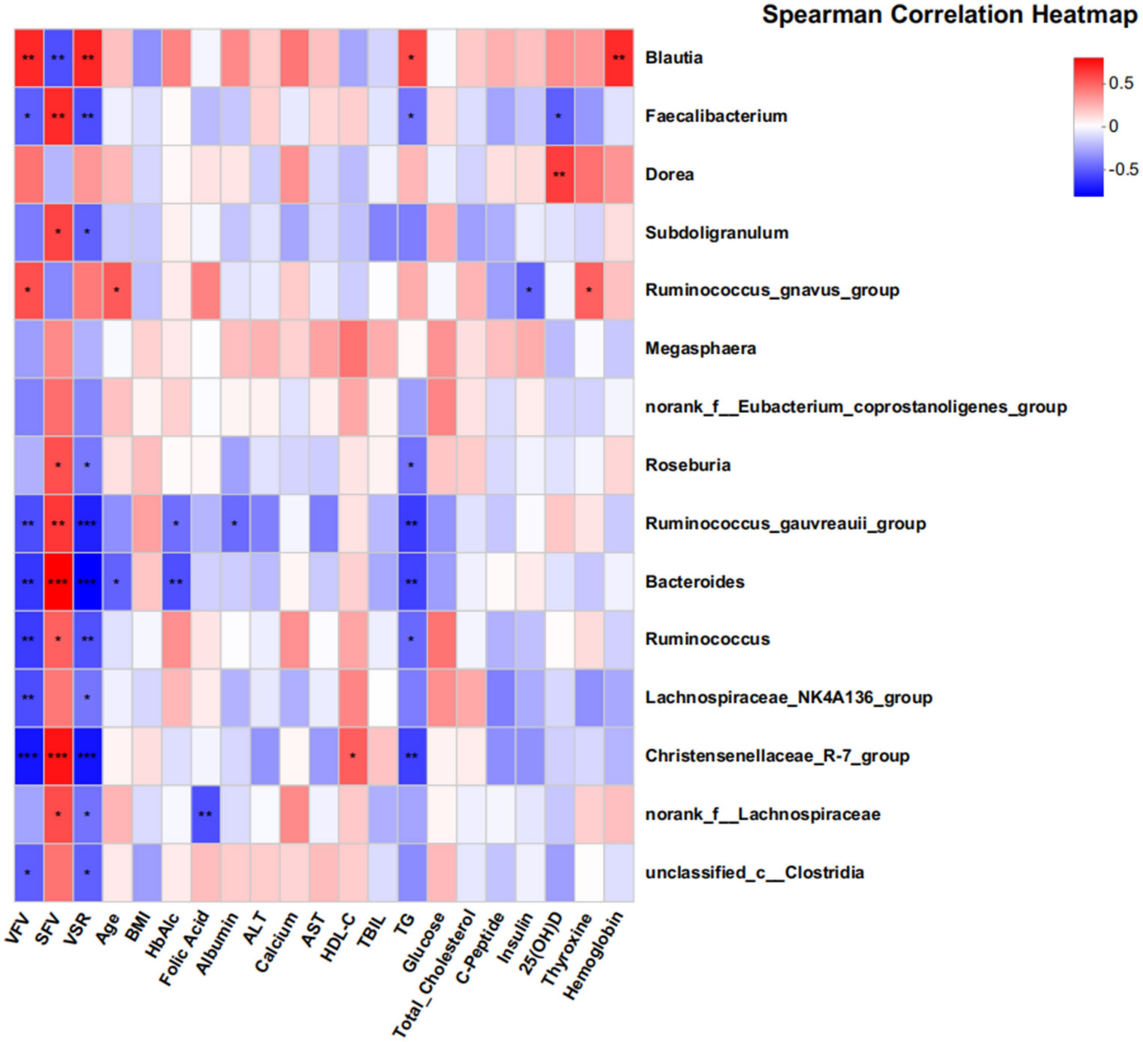
Associations between gut microbiota and clinical parameters in the discovery cohort. This heatmap displays partial Spearman correlation coefficients between the abundance of 18 genera and various clinical indices. The findings suggest significant associations between the gut microbiome and clinical parameters related to metabolic health. The color gradient reflects the strength and direction of the correlations, with red indicating positive correlations and blue indicating negative correlations. Statistical significance is denoted as follows: *p < 0.05; **p < 0.01; ***p < 0.001. Key clinical parameters include VFV, SFV, VSR, and TG.

Db -RDA at the genus level (Supplementary Figure 4) revealed that clinical parameters strongly associated with visceral obesity samples were VFV, total cholesterol, and TG, while SFV and HDL-C were more strongly associated with subcutaneous obesity samples. Figure 5 shows a Spearman correlation heatmap. *Blautia* was significantly positively correlated with VFV, SFV, VSR, and TG, suggesting its potential role in lipid metabolism and fat distribution. *Faecalibacterium* was negatively correlated with insulin and C-peptide levels, whereas Dorea showed a positive correlation with insulin levels. *Ruminococcus*, *Bacteroides*, and *Faecalibacterium* were negatively correlated with TG.

### 3.4 Role and predictive value of *Faecalibacterium* in visceral and subcutaneous obesity

**Figure 6:**
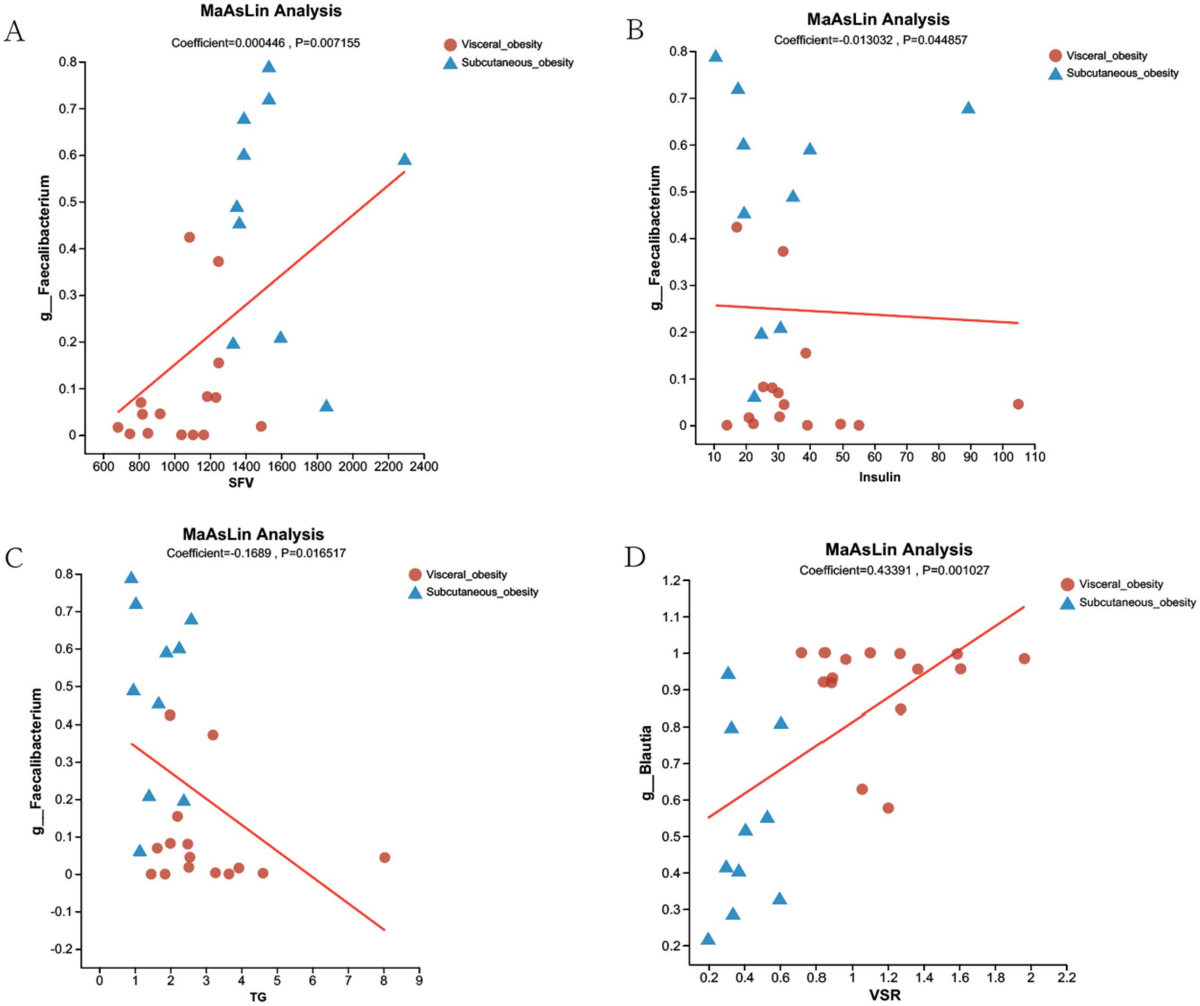
MaAsLin Analysis of Faecalibacterium and Blautia Associations with Clinical Parameters in Visceral and Subcutaneous Obesity. **(A)** MaAsLin analysis showing a positive correlation between *Faecalibacterium* abundance and SFV in both visceral obesity (red dots) and subcutaneous obesity (blue triangles) groups (Coefficient = 0.000446, *p* = 0.007155). **(B)** Negative correlation between *Faecalibacterium* abundance and insulin levels (Coefficient = −0.013032, *p* = 0.044857). **(C)** Negative correlation between *Faecalibacterium* abundance and TG (Coefficient = −0.1689, *p* = 0.016517). **(D)** Positive correlation between *Blautia* abundance and VSR (Coefficient = 0.43391, *p* = 0.001027). The red lines represent the regression lines showing the relationship between microbial abundance and the respective clinical parameters.

Differential species analysis and clinical correlation analysis revealed that *Blautia* and *Faecalibacterium* were associated with visceral and subcutaneous obesity, respectively, playing opposing roles. Given that *Blautia* has been studied previously, we selected *Faecalibacterium* for further analysis. Figure 6 explored the association of *Faecalibacterium* abundance with SFV, TG, and insulin levels using MaAsLin analysis. Figure 6A shows a positive correlation between *Faecalibacterium* abundance and SFV. Figures 6B,C indicate negative correlations between *Faecalibacterium* abundance and TG and insulin levels. Figure 6D demonstrates a significant positive correlation between *Blautia* abundance and VSR levels.

**Figure 7:**
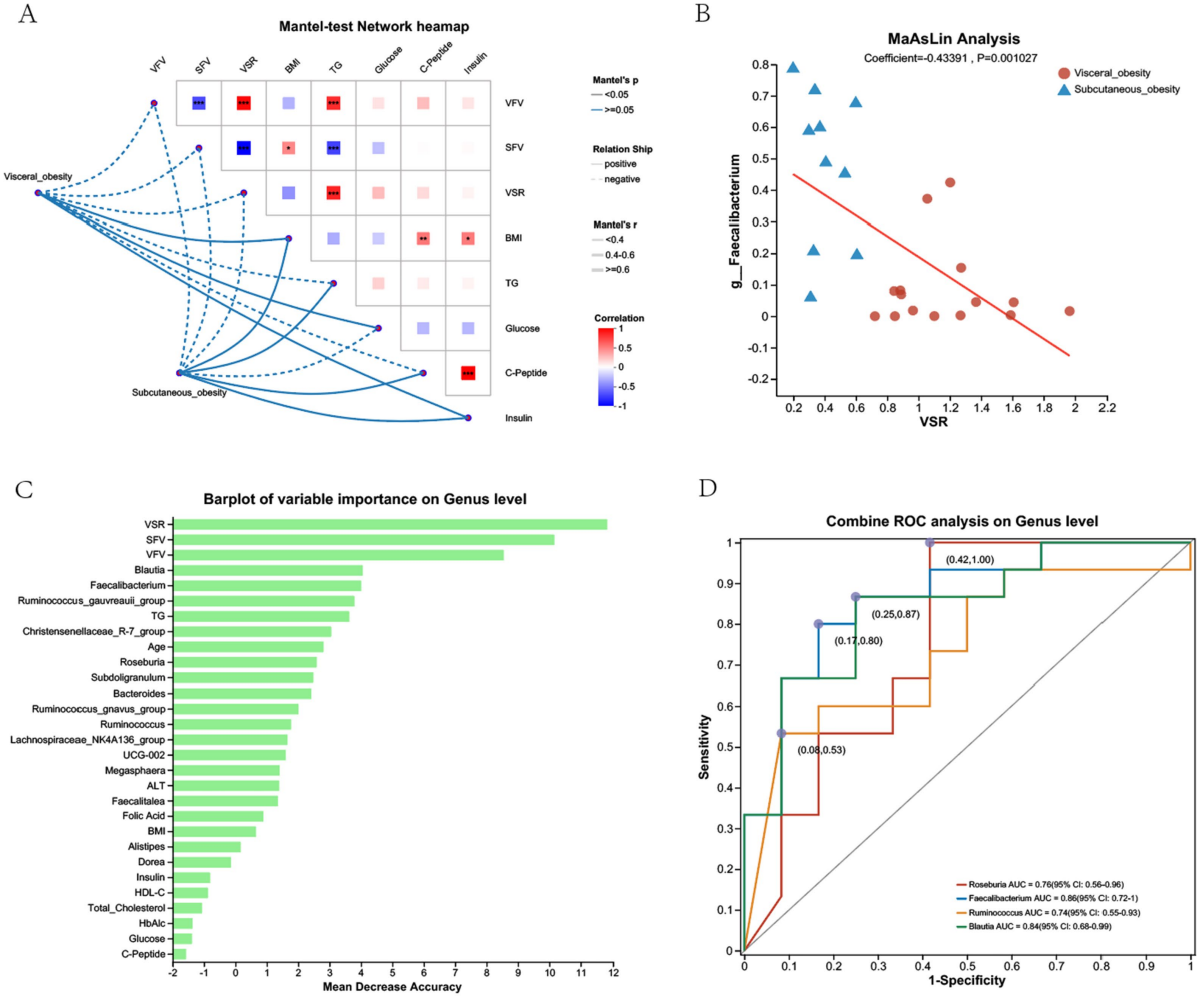
Correlation and predictive analysis of gut microbiota and metabolic parameters in the discovery cohort. **(A)** Mantel-test network heatmap showing the correlations between obesity-related clinical parameters (e.g., VFV, SFV, BMI) and gut microbiota composition at the genus level. The color intensity reflects the strength of the correlation, with red indicating positive correlations and blue indicating negative correlations. The dotted lines illustrate the network connections between visceral and subcutaneous obesity with clinical parameters. **(B)** MaAsLin Analysis displaying the relationship between the genus Faecalibacterium and VSR in visceral and subcutaneous obesity groups, with a positive correlation indicated by the red trend line. **(C)** Barplot of Variable Importance on Genus Level showing the mean decrease in accuracy for different genera in predicting obesity-related parameters. Higher values indicate greater importance in prediction accuracy. **(D)** Combined ROC Analysis on Genus Level illustrating the ROC curves for different genera, such as Roseburia, Faecalibacterium, Ruminococcus, and Blautia, in distinguishing between visceral and subcutaneous obesity. The AUC values with 95% confidence intervals are shown, indicating the diagnostic performance of each genus.

Figure 7 presents the correlation analysis between gut microbiota at the genus level and various obesity-related clinical parameters, along with the predictive value of specific genera in distinguishing visceral from subcutaneous obesity. Figure 7A shows a Mantel test network heatmap, where significant correlations were observed between VFV, SFV, and multiple genera. Figure 7B indicates a negative correlation between *Faecalibacterium* and VSR in both obesity groups, as shown by the red trend line in the MaAsLin analysis. Figure 7C displays the random forest importance rankings of different genera in predicting obesity-related parameters, showing that *Blautia* and *Faecalibacterium* had the highest predictive performance, aside from clinical factors like VFV, SFV, and VSR. Figure 7D presents ROC curves evaluating the diagnostic performance of various genera, including *Roseburia*, *Faecalibacterium*, *Ruminococcus*, and *Blautia*, in distinguishing visceral from subcutaneous obesity, with *Faecalibacterium* showing the highest AUC (0.86, CI: 0.72–1).

Supplementary Figure 5 shows the results of a combined ROC analysis at the genus level for distinguishing visceral and subcutaneous obesity. The combined ROC analysis of the top 7 negatively correlated genera yielded an AUC of 0.9 (95% CI: 0.77–1). Supplementary Figure 6 illustrates the combined ROC analysis of *Faecalibacterium* and TG levels for distinguishing visceral and subcutaneous obesity, showing that the combination reached an AUC of 0.87 (95% CI: 0.73–1).

### 3.5 Comprehensive analysis of functional potential and metabolic pathway differences in different types of obesity

**Figure 8:**
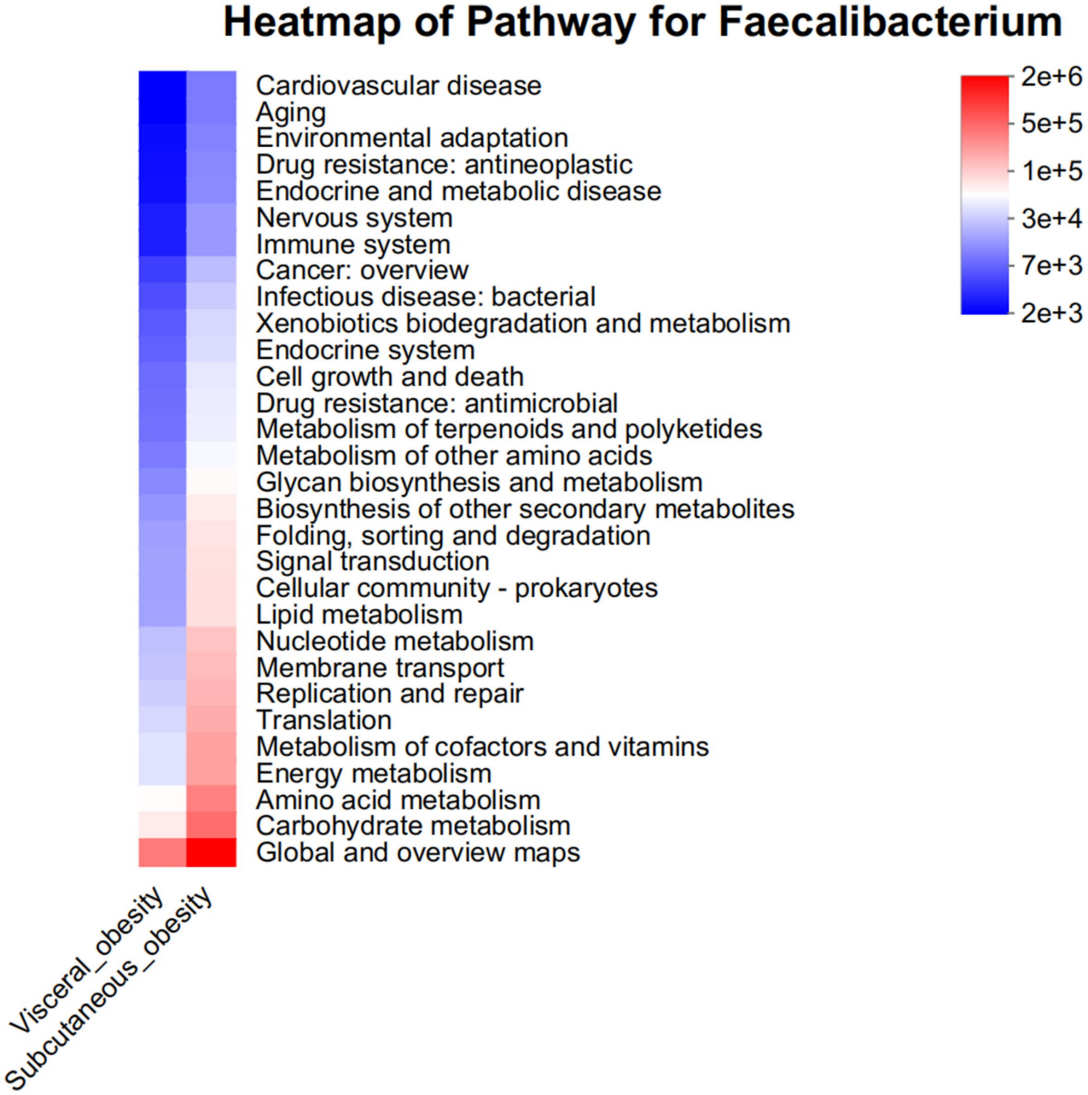
Pathway heatmap for *Faecalibacterium* in visceral and subcutaneous obesity in the discovery cohort. This heatmap displays the abundance of metabolic pathways associated with the Faecalibacterium genus in visceral obesity (red) and subcutaneous obesity (blue). The color intensity indicates the level of association with various pathways, including environmental adaptation, immune system response, carbohydrate metabolism, and lipid metabolism. This analysis highlights the distinct metabolic roles of Faecalibacterium in different obesity phenotypes.

Pathway analysis was conducted to explore the functional potential of the microbiota, as shown in Figure 8. This figure presents a heatmap of pathways at the *Faecalibacterium* genus level, indicating differences in functional gene content between the two obesity groups. Pathways related to carbohydrate metabolism and amino acid transport were particularly enriched in the visceral obesity group, suggesting that these microbes have distinct metabolic potential in this phenotype. Supplementary Figure 7 displays a bar chart of the predicted COG functional classification of *Faecalibacterium*, showing relatively consistent functional classification distribution between the two obesity groups, though there are slight differences in the proportions of specific functional categories. Supplementary Figure 8 also presents a heatmap of the predicted KEGG metabolic pathways for *Faecalibacterium*. The results indicate that, compared to the visceral obesity group, the subcutaneous obesity group is more active in carbohydrate metabolism, amino acid metabolism, energy metabolism, and cofactor and vitamin metabolism.

Supplementary Figures 8, 9 show the functional classification and metabolic pathway analysis results for the predicted functions of differential genera between visceral and subcutaneous obesity groups. The distribution of microbial functional classifications was very similar between the two groups, showing an even distribution across multiple functional categories.

### 3.6 Gut microbiota composition and differences between visceral and subcutaneous obesity in the validation cohort

Supplementary Figure 10A shows the relative abundance of gut microbiota at the genus level in patients with visceral obesity and subcutaneous obesity. The community barplot analysis reveals significant differences in the composition of gut microbiota between the visceral and subcutaneous obesity groups. Supplementary Figure 10B illustrates the differences in microbial abundance at the genus level between visceral and subcutaneous obesity groups, analyzed using the Wilcoxon rank-sum test. The abundance of *Faecalibacterium* and *Ruminococcus* was significantly higher in the subcutaneous obesity group compared to the visceral obesity group (*p* < 0.01), indicating that *Faecalibacterium* may also play an important role in distinguishing between obesity types in the validation cohort.

### 3.7 Differential expression of *Blautia* and *Faecalibacterium* in visceral and subcutaneous obesity

**Figure 9:**
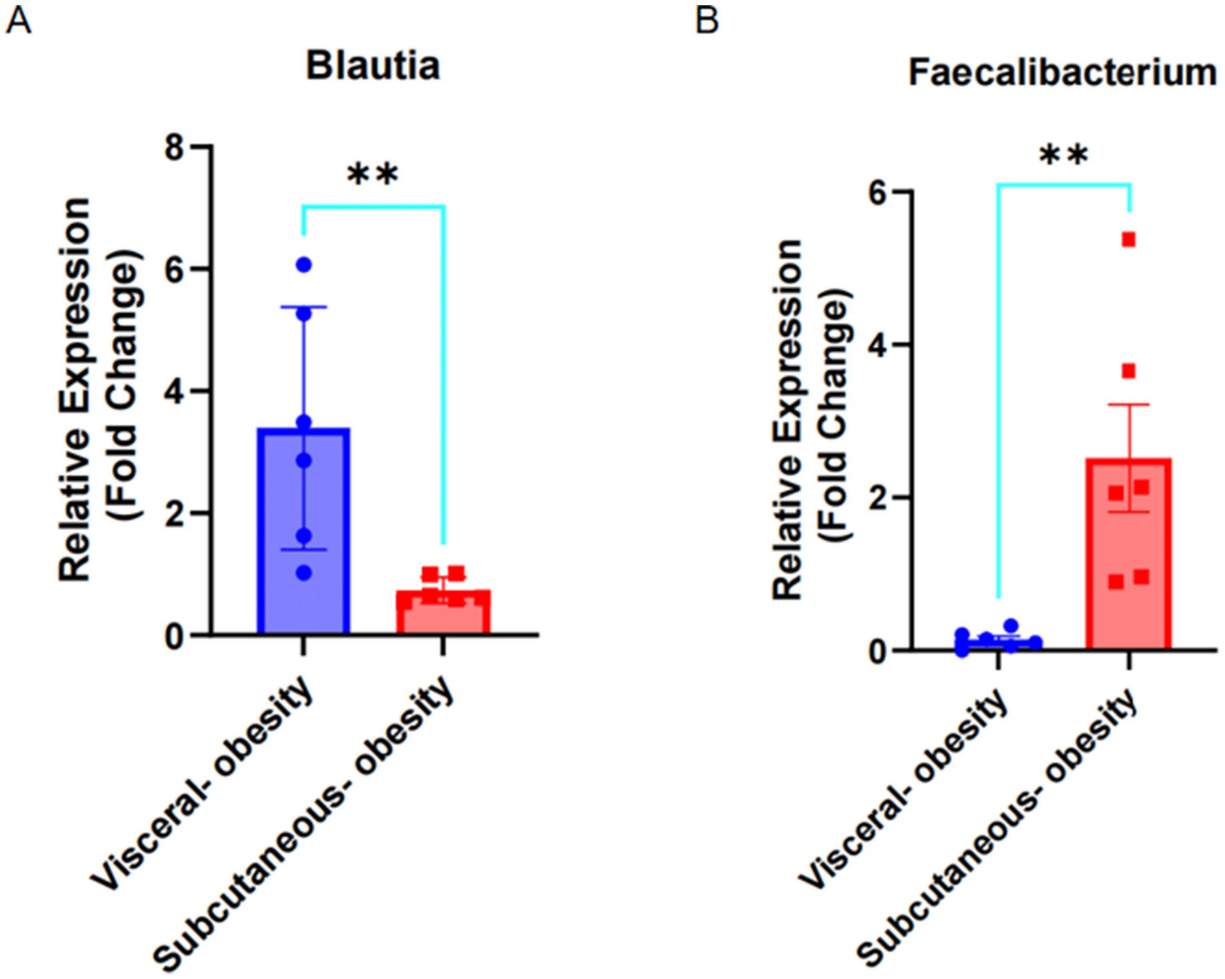
Expression of Blautia and Faecalibacterium in visceral obesity and subcutaneous obesity fecal samples. **(A)** Fold change of Blautia expression in visceral obesity (*n* = 6) and subcutaneous obesity (*n* = 6). **(B)** Fold change of Faecalibacterium expression in visceral obesity (*n* = 6) and subcutaneous obesity (*n* = 6).

The relative expression of *Blautia* and *Faecalibacterium* in fecal samples from visceral obese (*n* = 6) and subcutaneous obese (*n* = 6) individuals was evaluated using qPCR. As shown in Figure 9A, *Blautia* expression was significantly higher in visceral obesity compared to subcutaneous obesity (*p* < 0.01). In contrast, Figure 9B demonstrates that *Faecalibacterium* expression was significantly higher in subcutaneous obesity compared to visceral obesity (*p* < 0.01).

## 4 Discussion

Obesity is a multifactorial condition characterized by excessive body fat accumulation and associated with numerous metabolic disorders. Among various types of body fat, visceral fat stored around internal organs within the abdominal cavity has been identified as a critical risk factor for metabolic diseases, including cardiovascular disease, type 2 diabetes, and certain cancers. Unlike subcutaneous fat, visceral fat is metabolically active and contributes to the dysregulation of metabolic and inflammatory pathways.

In terms of fat distribution measurement, traditional anthropometric parameters such as BMI, WC, and waist-to-hip ratio do not directly reflect the amount and distribution of body fat. CT and MRI are considered the gold standards for measuring visceral fat content (Gu et al., 2020; Li et al., 2022). Compared to MRI, which is expensive and time-consuming, CT is relatively inexpensive, non-invasive, fast, and less affected by respiratory artifacts (Fang et al., 2018). At the same time, CT can clearly distinguish different body tissue components, independently measure the amount of visceral and subcutaneous abdominal fat, and has high reproducibility (Kim et al., 2022; Gu et al., 2020; Park et al., 2020). All clinically obese patients we collected routinely underwent abdominal CT after hospitalization, so using opportunistic CT to measure visceral and subcutaneous fat is both convenient and accurate without adding an extra burden to the patients.

Previous research reports on visceral fat measurement mostly focused on measuring the area at a certain level, with the Japanese Obesity Research Society proposing an absolute value of 100 cm^2^ as a clinical reference for visceral obesity to assess the risk of obesity-related diseases (Obesity ECoCfODiJJSftSo, 2002). Although the studies used CT methods for measurement, they used thick-slice images, making the measurements potentially inaccurate. Additionally, fat distribution varies between individuals based on factors like genetics, gender, and lifestyle, which are not reflected by measuring only visceral fat area. VFV of more than 100 cm^2^ is often used as a threshold for visceral obesity, but it does not fully capture the complexity of fat distribution throughout the body (Shuster et al., 2012). Regardless of the total fat, it is not accurate to define visceral obesity just according to the visceral fat area.

Accurate measurement of visceral fat is essential for assessing its impact on health. CT scans are widely regarded as the gold standard for quantifying visceral fat. CT imaging allows precise differentiation between visceral and subcutaneous fat, enabling measurement of visceral fat area, volume, and the VSR. These metrics provide valuable insights into the distribution and extent of visceral fat, facilitating the assessment of its role in metabolic dysfunction. The percentage of visceral fat or subcutaneous fat or VSR should be the ideal evaluation of fat distribution. However, there is no way to evaluate the weight of total fat, visceral fat, or subcutaneous fat by current technology. Therefore, we estimate the fat distribution by fat area or volume instead of fat weight. To improve the accuracy of fat distribution measurements, we have controlled the BMI range as closely as possible to minimize variations and better reflect the differences in fat distribution.

VSR is an important measure, with a higher VSR indicating greater visceral fat relative to subcutaneous fat, which can better represent the fat distribution. Elevated VSR is associated with a higher risk of obesity-related health complications (Fox et al., 2007). Our study investigated the difference in the gut microbiota between high VSR and low VSR calculated by the volume of visceral and subcutaneous fat in the L3 vertebral region in obese patients measured by CT. This study is supposed to find specific microbial signatures predictive of visceral fat accumulation and microbiota-targeted therapies which can reduce visceral fat, improve metabolic health, and mitigate the risks associated with excess visceral fat.

Our results showed *Faecalibacterium*, *Subdoligranulum*, *Bacteroides*, *Megamonas*, *Megasphaera*, *Roseburia*, and *Ruminococcus* were more abundant in the subcutaneous obesity group, while *Blautia*, *Dorea*, and *Ruminococcus_gnavus_group* were more associated with visceral obesity. *Bacteroides*, *Faecalibacterium*, *Ruminococcus, Subdoligranulum*, and *Megasphaera* are rich in members in Asians (Syromyatnikov et al., 2022). All these above differential microbiomes except *Subdoligranulum* are associated with obesity and metabolic diseases, which also showed our results are reliable. The genera of Bacteroides, *Faecalibacterium*, and *Roseburia* were negatively associated with T2D, while the genera of *Ruminococcus*, and *Blautia* were positively associated with T2D (Gurung et al., 2020).

*Subdoligranulum* is less well-studied and there is no report about the association between *Subdoligranulum* and obesity. *Bacteroides* are abundant in the gut and are involved in breaking down complex carbohydrates. Imbalances in *Bacteroides* have been associated with obesity and metabolic diseases (Wu et al., 2023; Bai et al., 2023). *Bacteroides* were also showed negatively associated with advanced steatosis (Dong et al., 2021). *Megamona*s are involved in fermenting amino acids and can influence the production of SCFAs, which affect energy balance and fat storage (Wu et al., 2024). *Megasphaeras* were positively associated with Obesity (Kang et al., 2019) and steatosis (Dong et al., 2021). Similar to *Megamonas*, these bacteria contribute to SCFA production and may impact obesity through their metabolic effects.

*Ruminococcus* were obesity-associated genera in studies from the West but lean-associated in the East (Xu et al., 2022). *Roseburia* are Known for their role in producing SCFAs like butyrate, which has anti-inflammatory properties and can influence fat storage and metabolism (Zeng et al., 2019; Tamanai-Shacoori et al., 2017; Kaminska et al., 2022). The *Faecalibacterium* genus is one of the most abundant bacteria in the gut microbiota of healthy adults and one of the six taxa most strongly correlated with glucose metabolism (Miquel et al., 2013). *Faecalibacterium* produces butyrate, a major fermentation product for these taxa (e.g., after dietary fiber degradation), and an SCFA that has been shown to affect fasting and postprandial glucose metabolism through various mechanisms (Palmnäs-Bédard et al., 2022). It is reported that the Faecalibacterium can increase muscle mass improve hepatic health, and decrease adipose tissue inflammation in mice (Munukka et al., 2017). Our results showed that Faecalibacterium has a higher expression in the high VSR (visceral-to-subcutaneous fat ratio) group. It can well predict the visceral-to-subcutaneous fat ratio and is a good Gut Microbiota marker for visceral obesity diagnosis. Our results showed many previously reported obesity-related gut Microbiota are also altered during different visceral-to-subcutaneous fat ratio.

To better define the fat distribution, we choose patients with similar BMI between 35 and 40, which is an advantage of our research but also the limitation of this study. Next, we will augment the population and BMI range to confirm whether the differential gut microbiota is consistent in different BMI ranges. Although some differential gut microbiota like *Bacteroides*, *Faecalibacterium*, *Ruminococcus*, *Subdoligranulum*, and *Megasphaera* are reported they are associated with obesity, whether they play roles and the mechanism in fat distribution is not known. *Faecalibacterium* is a potential gut microbiota biomarker for the high VSR population. More clinic populations and investigations are required to confirm and optimize it as a potential gut microbiota diagnosis.

Now we do not know whteher the gut microbiota that differ in the faces of participants with different types of obesity is cause or results. Maybe it is the results since different types of obesity have different genotype or different dietary style which results in different gut micro-environment for different germ. Maybe Faecalibacterium is more suitable for gut environment in subcutaneous obesity. Also, maybe *Faecalibacterium* also can affect obesity type. Now many reports showed gut microbiota can adjust obesity by its metabolites. SCFAs is the most examined intestinal microbial metabolites. Among SCFAs, Acetate, propionate, and butyrate are more than 95%. SCFAs come from non-digestible host dietary fber fermented by the gut microbiota. SCFAs can serve as energy substrates and infuence the maturation of microglia in the CNS once absorbing into the circulation (Coppola et al., 2021). *Faecalibacterium prausnitzii* is a main butyrate producer (van Deuren et al., 2022). Butyrate promotes the conversion of white adipose tissue (WAT) to beige adipose tissue by activating AMPK (adenosine monophosphate activated protein kinase) and PGC-1 *α* (peroxisome proliferator activated receptor gamma co activator 1α), increasing heat production and energy expenditure. Butyrate reduces fat production by inhibiting HDAC, downregulating the expression of PPAR *γ* (peroxisome proliferator activated receptor γ) and SREBP-1c (steroid regulatory element binding protein 1c). Butyrate activates G protein coupled receptors (such as GPR41/GPR43), increases the activity of hormone sensitive lipase (HSL), and promotes triglyceride hydrolysis. Butyrate stimulates intestinal L cells to secrete glucagon like peptide-1 (GLP-1), improving insulin sensitivity and reducing visceral fat accumulation. Butyrate reduces inflammatory factors (such as TNF-α and IL-6) in adipose tissue and improves insulin resistance by inhibiting the NF-kB pathway. Butyrate is the main energy source for colonic epithelial cells, which enhances the expression of tight junction proteins such as occludin and claudin, reduces the entry of endotoxins (LPS) into the bloodstream, and lowers systemic inflammation and fat accumulation. Butyrate, as an HDAC inhibitor, regulates the expression of fat metabolism related genes (such as FGF21 and adiponectin) through histone acetylation, affecting fat distribution. Butyrate may regulate appetite related neurons (such as POMC neurons) in the hypothalamus through the blood–brain barrier or vagus nerve signaling, reducing feeding behavior. Butyrate tends to reduce visceral fat (strongly associated with metabolic disease risk) rather than subcutaneous fat, possibly due to its high content in the intestine and portal vein, as well as promoting fatty acid oxidation in the intestine and viscera. Butyrate regulates fat distribution through multiple targets and pathways, with core mechanisms including promoting fat browning, inhibiting fat synthesis, improving insulin sensitivity, anti-inflammatory and gut microbiota regulation, and fat distribution. These effects make it a potential molecule for combating obesity and metabolic syndrome, but further research is needed for specific clinical applications (van Deuren et al., 2022; Wang et al., 2025).

Several limitations should be acknowledged in this study. First, the absence of a healthy control group prevents direct comparison of microbial abundance between obese and healthy populations, though our findings regarding the differential distribution of Faecalibacterium between obesity subtypes still provide novel insights into microbial heterogeneity. Second, the functional predictions derived from PICRUSt2, while generating valuable hypotheses, remain speculative due to their reliance on phylogenetic marker gene inference rather than direct genomic evidence. These predicted functional profiles should be interpreted with caution and validated through targeted metagenomic sequencing or metabolomic analyses in future studies. Third, due to the difficulty of the sample collection, the sample size is limited, which both groups are around 15. However, it is enough to analyze and provide a reliable results. Also we did qPCR to futher checked our results and found the results is repeatable by qPCR.

In conclusion, We find differential gut microbiota in different VSR groups. *Faecalibacterium* can be a good gut microbiota biomarker for a high VSR population, which provides possible economic, non-radiation, and invasion methods for evaluating fat distribution and visceral obesity. Also, Managing the composition of gut microbiota through diet, probiotics, or other interventions may help address visceral obesity and improve overall metabolic health.

## Data Availability

The raw sequencing data is deposited in the NCBI Sequence Read Archive (SRA) under the BioProject accession number PRJNA1309258. The data are publicly available at: https://www.ncbi.nlm.nih.gov/bioproject/PRJNA1309258.

## Footnotes

^1^ https://cloud.majorbio.com

^2^ http://huttenhower.sph.harvard.edu/LEfSe
