# Supplementary material for: Identifying gut microbiota Faecalibacterium as a potential biomarker for distinguishing visceral or subcutaneous obese population
Source: Front Microbiol. 2025 Aug 29;16:1635962. doi: 10.3389/fmicb.2025.1635962 (PMC12426024; doi:10.3389/fmicb.2025.1635962)
Supplement: Supplementary file 1 [file Supplementary_file_1.docx]

**Supplementary Content**

***Figures***

**Supplementary Figure 1.** Flowchart of Participant Enrollment.

**Supplementary Figure 2.** Comparison of Gut Microbiota Diversity Between Visceral and Subcutaneous Obesity**.**

**Supplementary Figure 3.** Taxonomic Differences Between Visceral and Subcutaneous Obesity at Various Taxonomic Levels.

**Supplementary Figure 4.** Genus-level db-RDA Analysis Showing the Association Between Clinical Parameters in Visceral and Subcutaneous Obesity Groups.

**Supplementary Figure 5.** Combined ROC Analysis at the Genus Level for Differentiating Obesity Types Based on Top 7 Negative Genera and Triglycerides.

**Supplementary Figure 6.** Combined ROC Analysis at the Genus Level for Differentiating Obesity Types Based on *Faecalibacterium* and Triglycerides.

**Supplementary Figure 7.** COG Function Classification for *Faecalibacterium* in Visceral and Subcutaneous Obesity.

**Supplementary Figure 8.** Heatmap of Pathway Analysis for All Genera in Visceral and Subcutaneous Obesity.

**Supplementary Figure 9.** The functional classification and metabolic pathway analysis of the gut microbiota in the visceral obesity and subcutaneous obesity groups.

**Supplementary Figure 10.** Gut microbiota composition and differences between visceral and subcutaneous obesity in the validation cohort.

***Table***

**Supplementary Table 1.** Baseline characteristics of participants in the validation cohort.


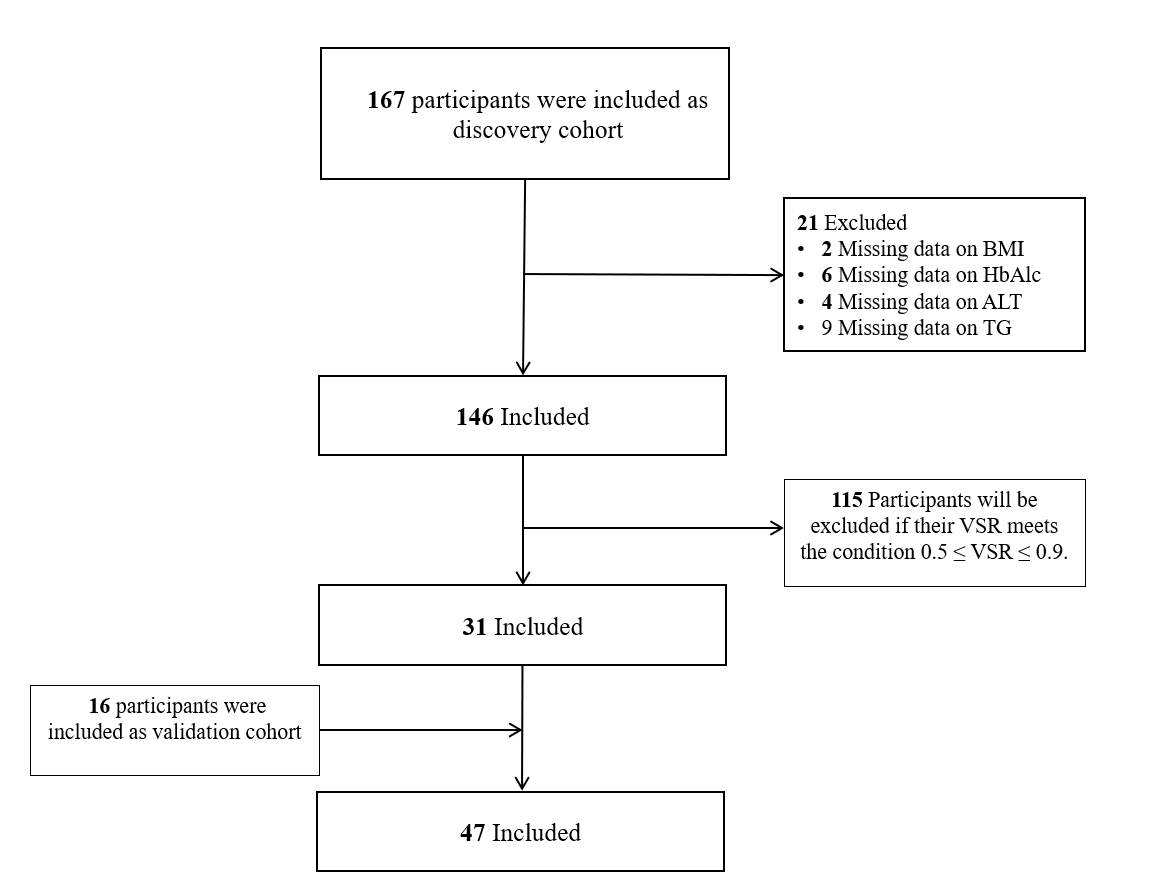


**Supplementary Figure 1.** Flowchart of Participant Enrollment.


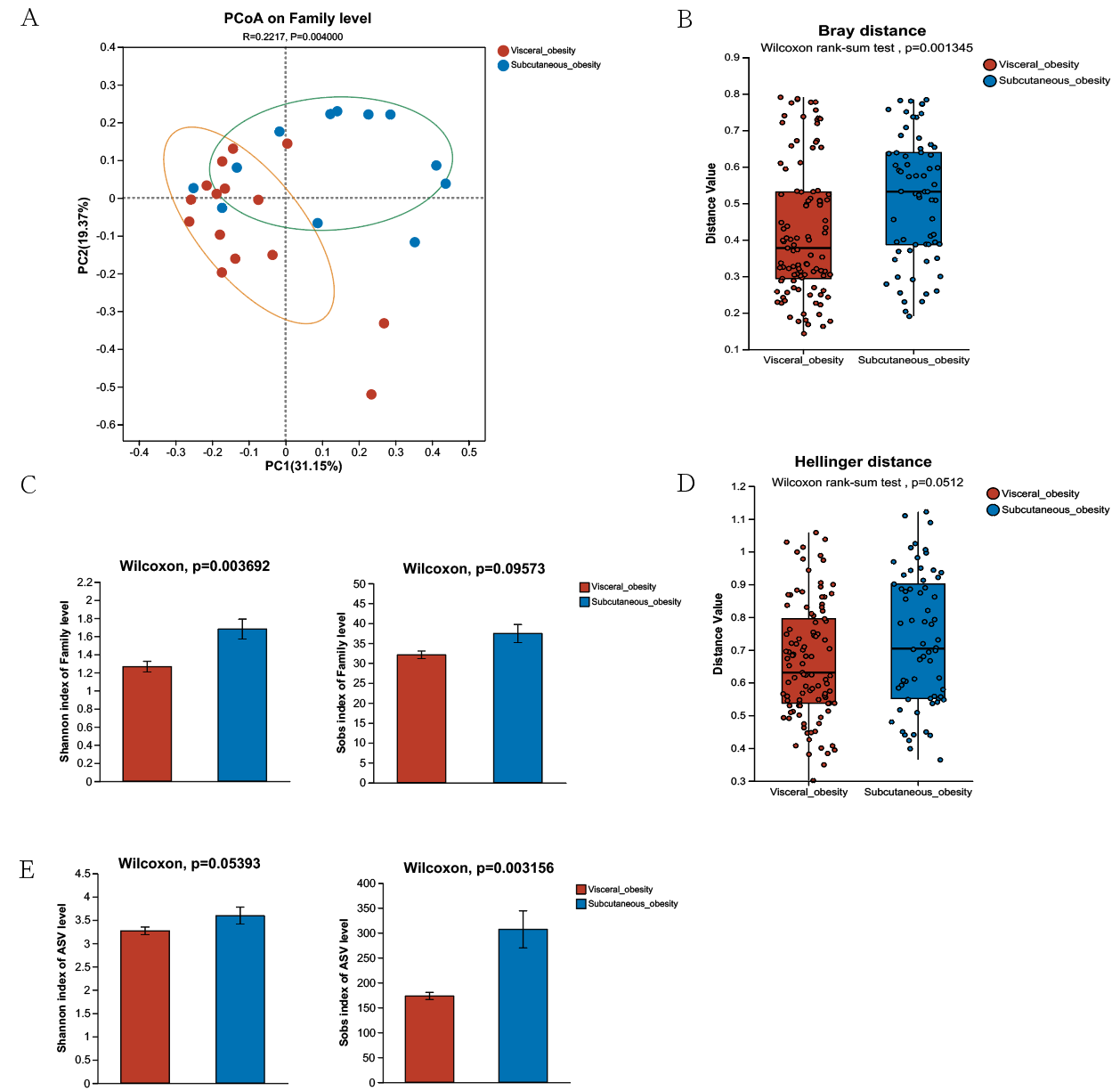


**Supplementary Figure 2.** Comparison of Gut Microbiota Diversity Between Visceral and Subcutaneous Obesity.

(A) PCoA based on family-level taxonomic composition, showing the separation of microbiota between visceral obesity (red) and subcutaneous obesity (blue). The variation explained by PC1 (31.15%) and PC2 (19.37%) is displayed, with R² = 0.2217 and *P* = 0.004.

(B) Boxplot comparing Bray-Curtis distances between visceral and subcutaneous obesity groups (*P* = 0.001345).

(C) The Shannon index shows a significant difference between groups (*P* = 0.003692), while the Sob index is not statistically significant (*P* = 0.09573).

(D) Boxplot comparing Hellinger distances between the two obesity groups (*P* = 0.0512).


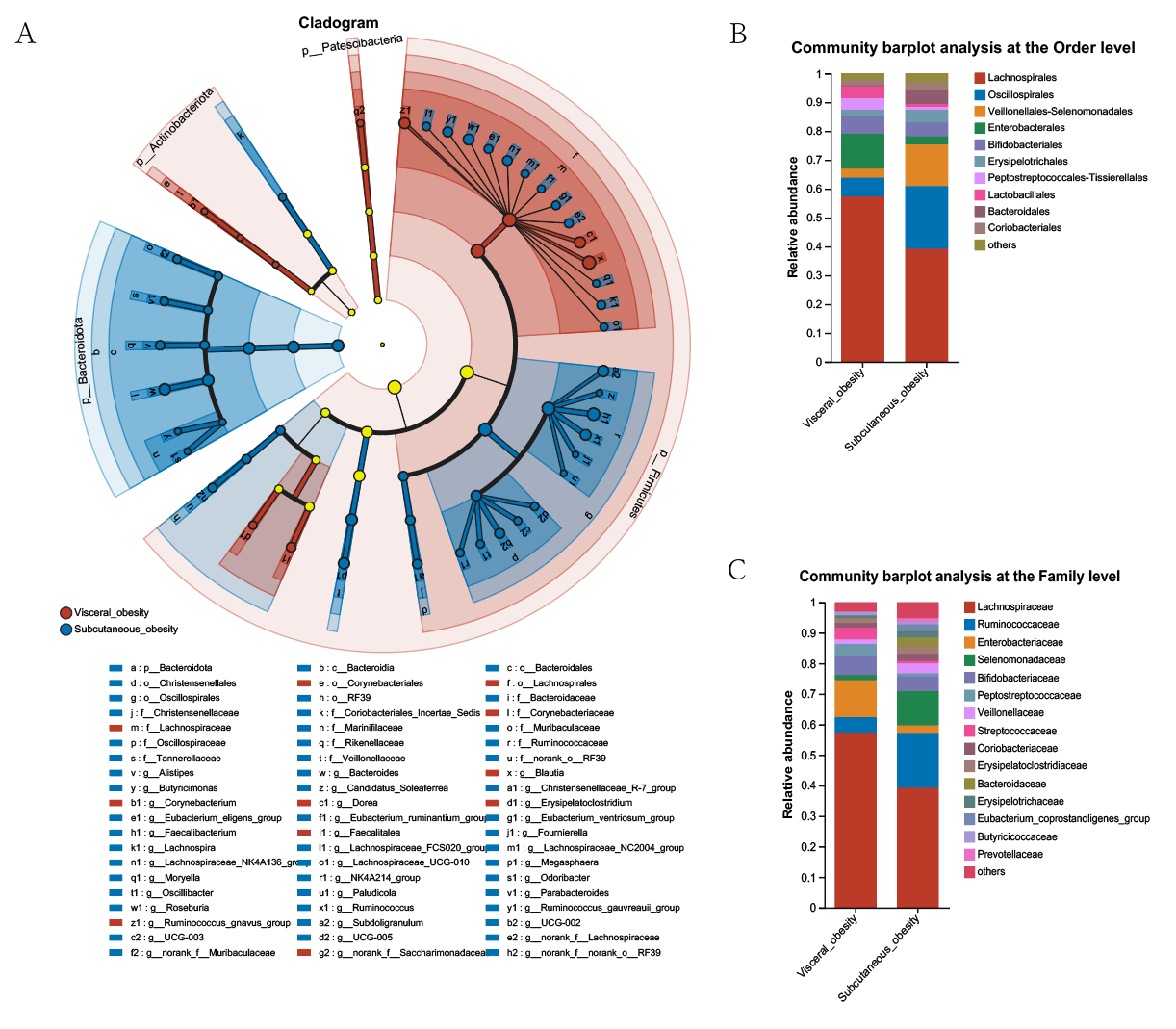


**Supplementary Figure 3.** Taxonomic Differences Between Visceral and Subcutaneous Obesity at Various Taxonomic Levels.

(A) Cladogram displaying the phylogenetic distribution of microbial taxa associated with visceral obesity (red) and subcutaneous obesity (blue). Taxa enriched in each group are highlighted, with significant differences observed at multiple taxonomic levels, including phylum, class, and family.

(B) Community bar plot analysis at the order level, showing the relative abundance of various microbial orders in visceral and subcutaneous obesity groups. *Lachnospirales*, , and *Oscillospirales* are among the most dominant orders in both groups, with varying proportions.

(C) Community bar plot analysis at the family level, illustrating the distribution of microbial families. The visceral obesity group exhibits a higher relative abundance of *Lachnospiraceae*, while the subcutaneous obesity group shows greater representation of *Ruminococcaceae*.


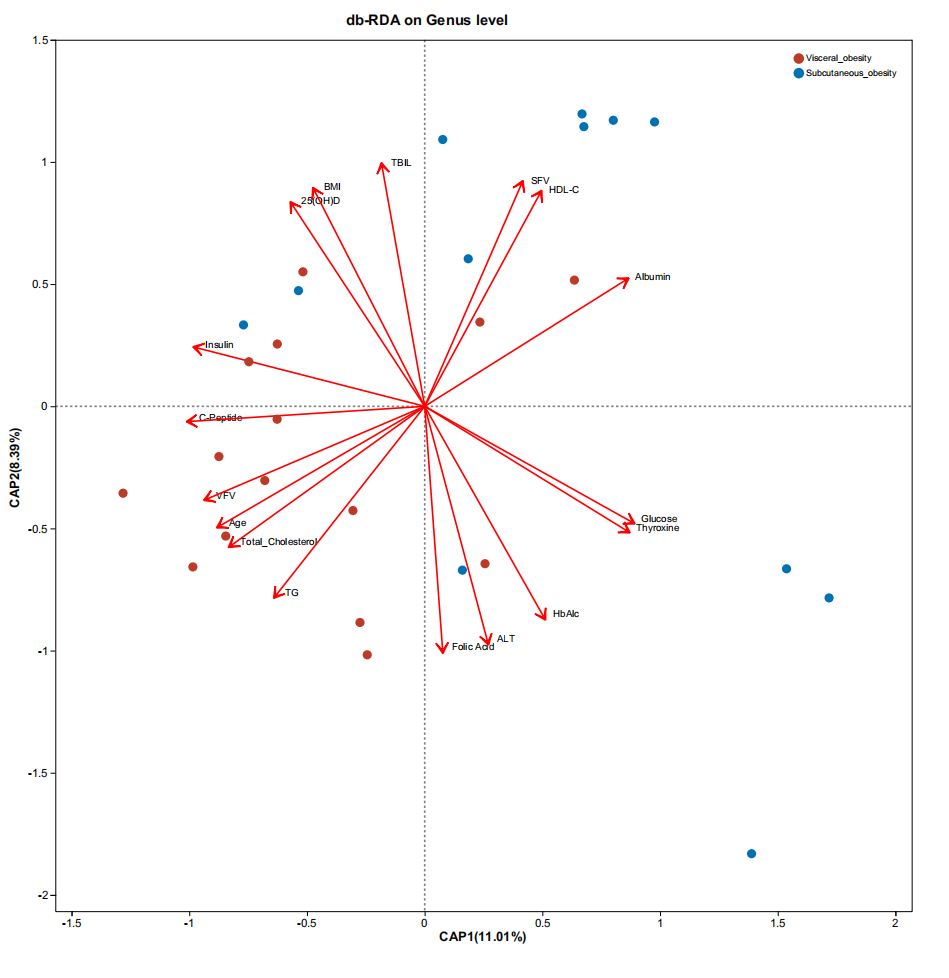
**Supplementary Figure 4.** Genus-level db-RDA Analysis Showing the Association Between Clinical Parameters in Visceral and Subcutaneous Obesity Groups. This figure presents a distance-based redundancy analysis (db-RDA) at the genus level, illustrating the associations between microbial communities and clinical parameters in visceral obesity (red dots) and subcutaneous obesity (blue dots) groups. The red arrows indicate the direction and strength of each clinical parameter. The visceral obesity group is more closely associated with VFA, total cholesterol, triglycerides (TG), and insulin, while the subcutaneous obesity group shows a stronger correlation with subcutaneous fat area (SFA) and high-density lipoprotein cholesterol (HDL-C).


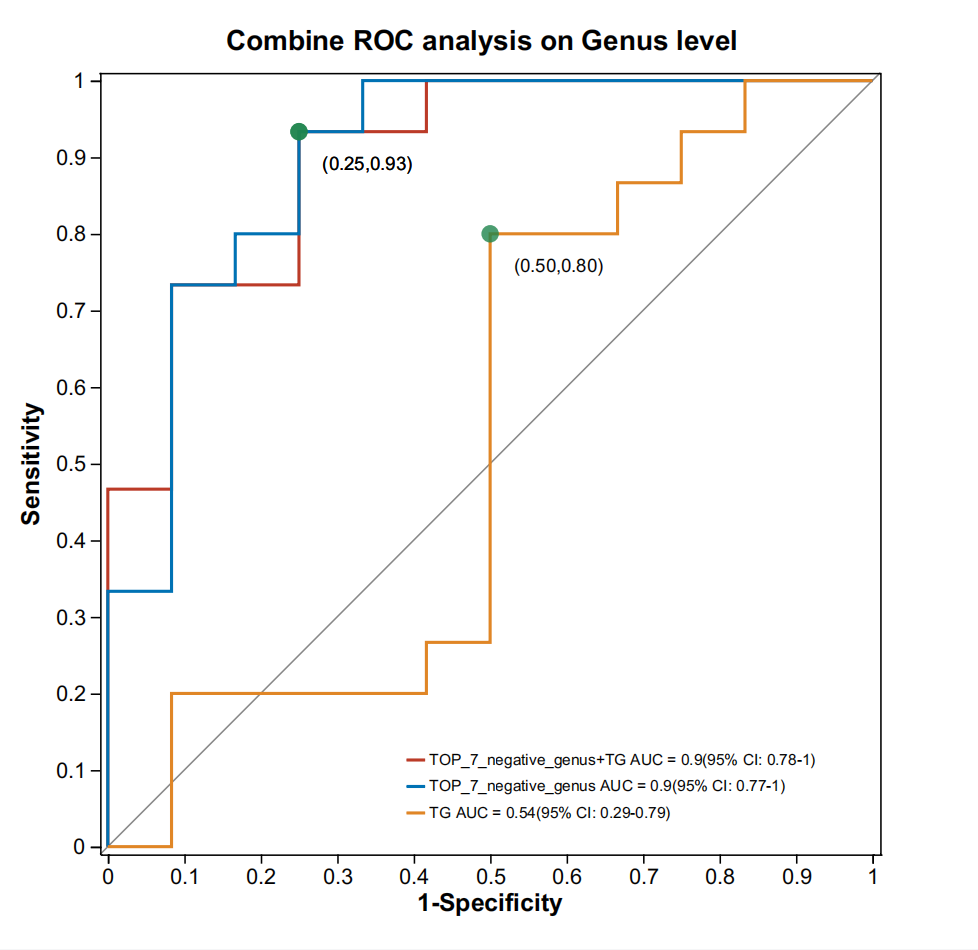


**Supplementary Figure 5.** Combined ROC Analysis at the Genus Level for Differentiating Obesity Types Based on Top 7 Negative Genera and Triglycerides.

The figure shows the results of a combined ROC analysis at the genus level, used to distinguish between visceral and subcutaneous obesity. The blue curve represents the ROC analysis of the top 7 genera negatively correlated with TG levels, with an AUC of 0.9 (95% CI: 0.77-1). The orange curve represents the ROC analysis of TG levels, with an AUC of 0.54 (95% CI: 0.29-0.79). The red curve shows the combined ROC analysis of the top 7 genera and TG levels, with an AUC of 0.9 (95% CI: 0.78-1).


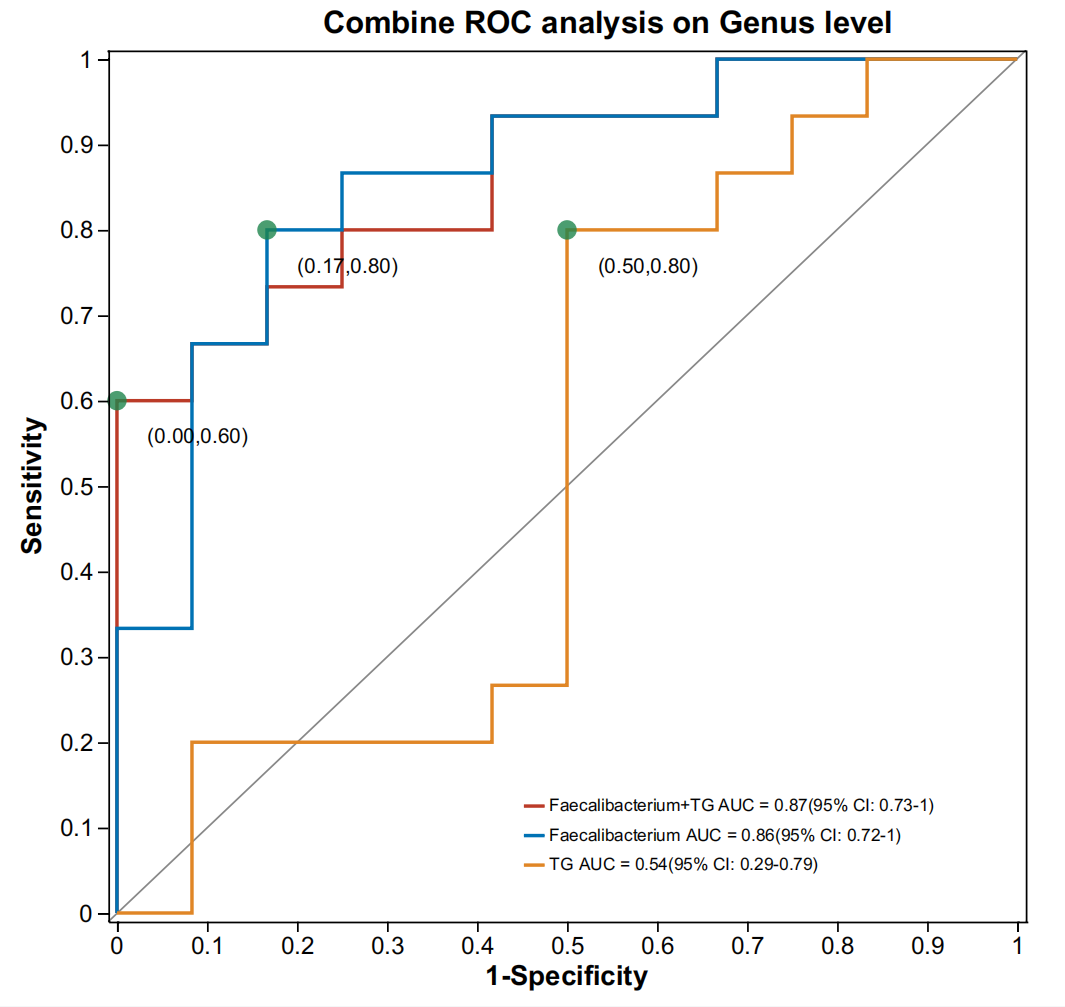


**Supplementary Figure 6.** Combined ROC Analysis at the Genus Level for Differentiating Obesity Types Based on Faecalibacterium and Triglycerides.

The figure presents the combined ROC analysis results of *Faecalibacterium* and TG levels in distinguishing visceral and subcutaneous obesity. The blue curve represents the ROC analysis of *Faecalibacterium*, with an AUC of 0.86 (95% CI: 0.72-1). The orange curve represents the ROC analysis of TG levels, with an AUC of 0.54 (95% CI: 0.29-0.79). The red curve shows the combined ROC analysis of *Faecalibacterium* and TG levels, with an AUC of 0.87 (95% CI: 0.73-1).


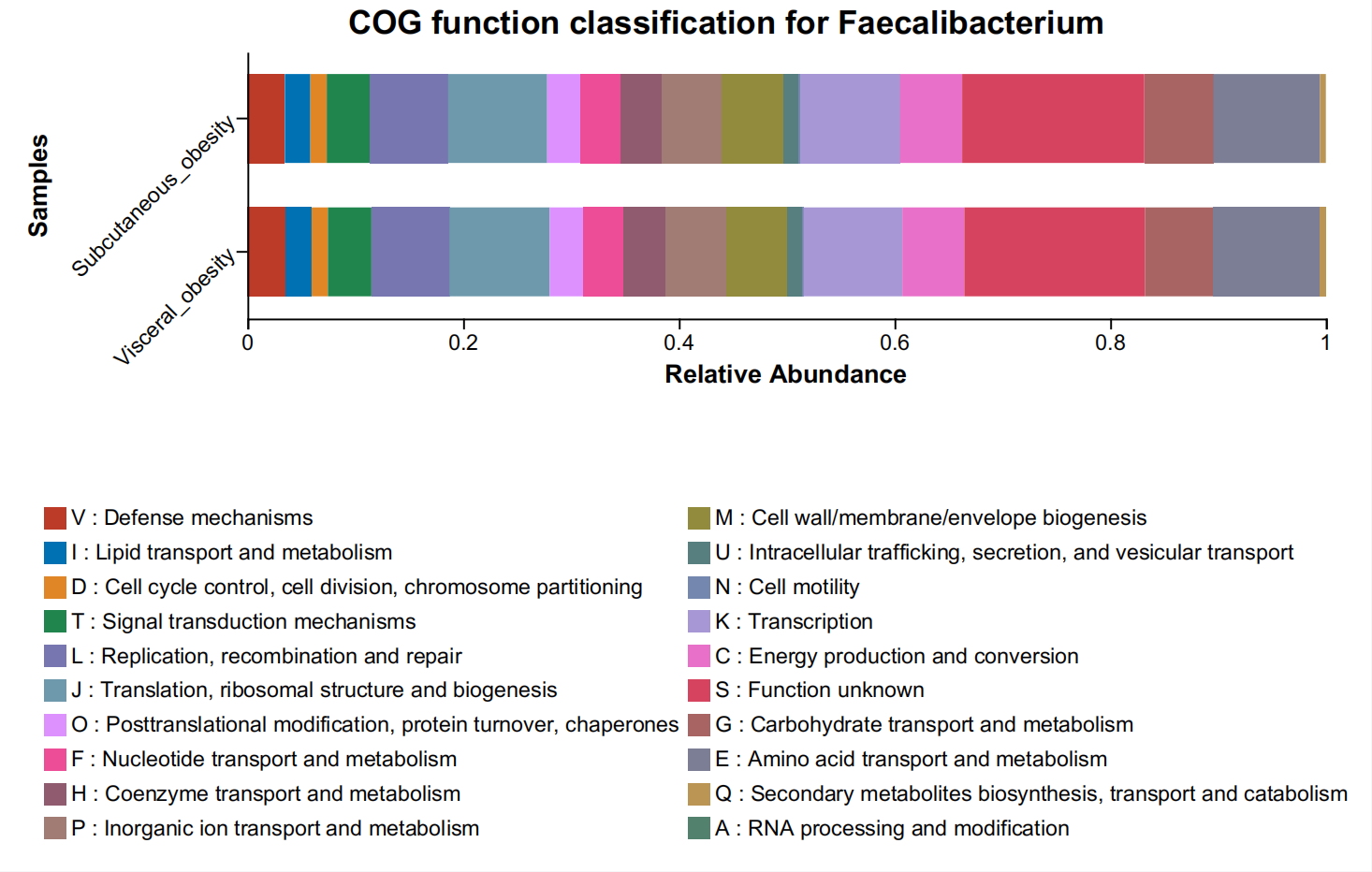


**Supplementary Figure 7.** COG Function Classification for *Faecalibacterium* in Visceral and Subcutaneous Obesity.

The bar plot displays the COG function classification for *Faecalibacterium* in visceral obesity (bottom) and subcutaneous obesity (top) groups, based on relative abundance.


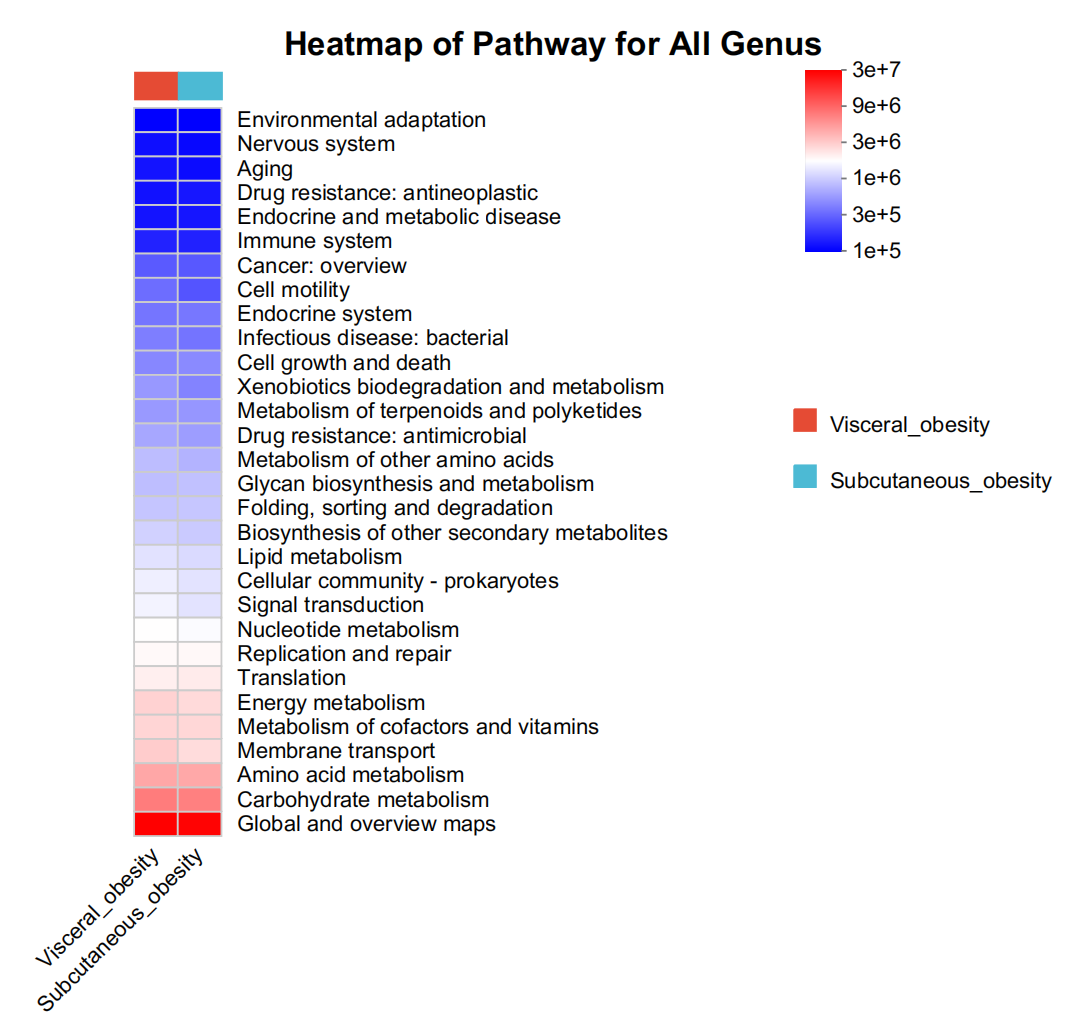


**Supplementary Figure 8.** Heatmap of Pathway Analysis for All Genera in Visceral and Subcutaneous Obesity.

The heatmap illustrates the predicted functional pathways for all bacterial genera in the visceral obesity (red) and subcutaneous obesity (blue) groups. Pathways are categorized by their biological functions, such as lipid metabolism, carbohydrate metabolism, amino acid metabolism, and immune system processes.


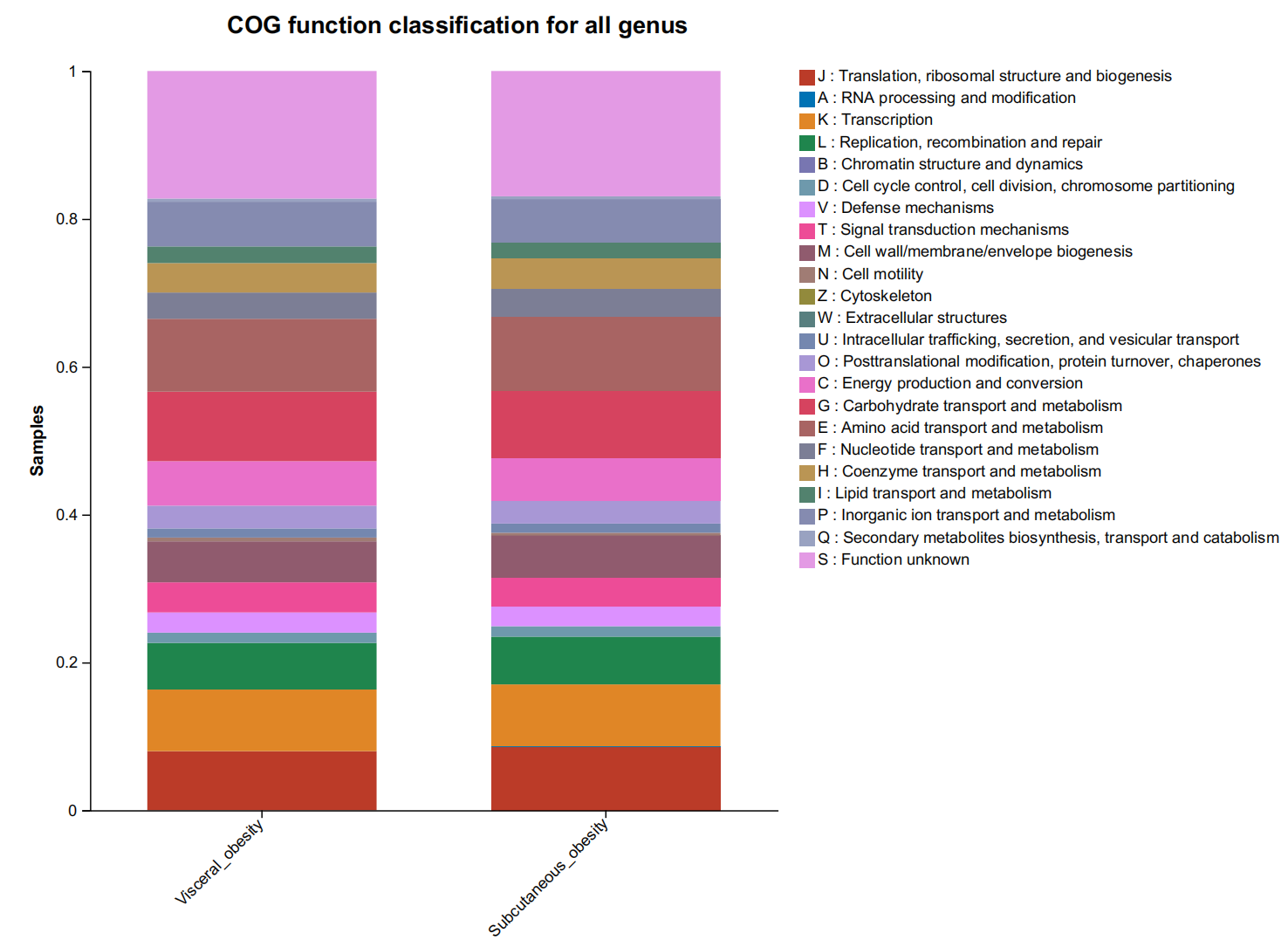


**Supplementary Figure 9. The functional classification and metabolic pathway analysis of the gut microbiota in the visceral obesity and subcutaneous obesity groups.** The bar plot illustrates the relative abundance of various COG functional categories across all genera.


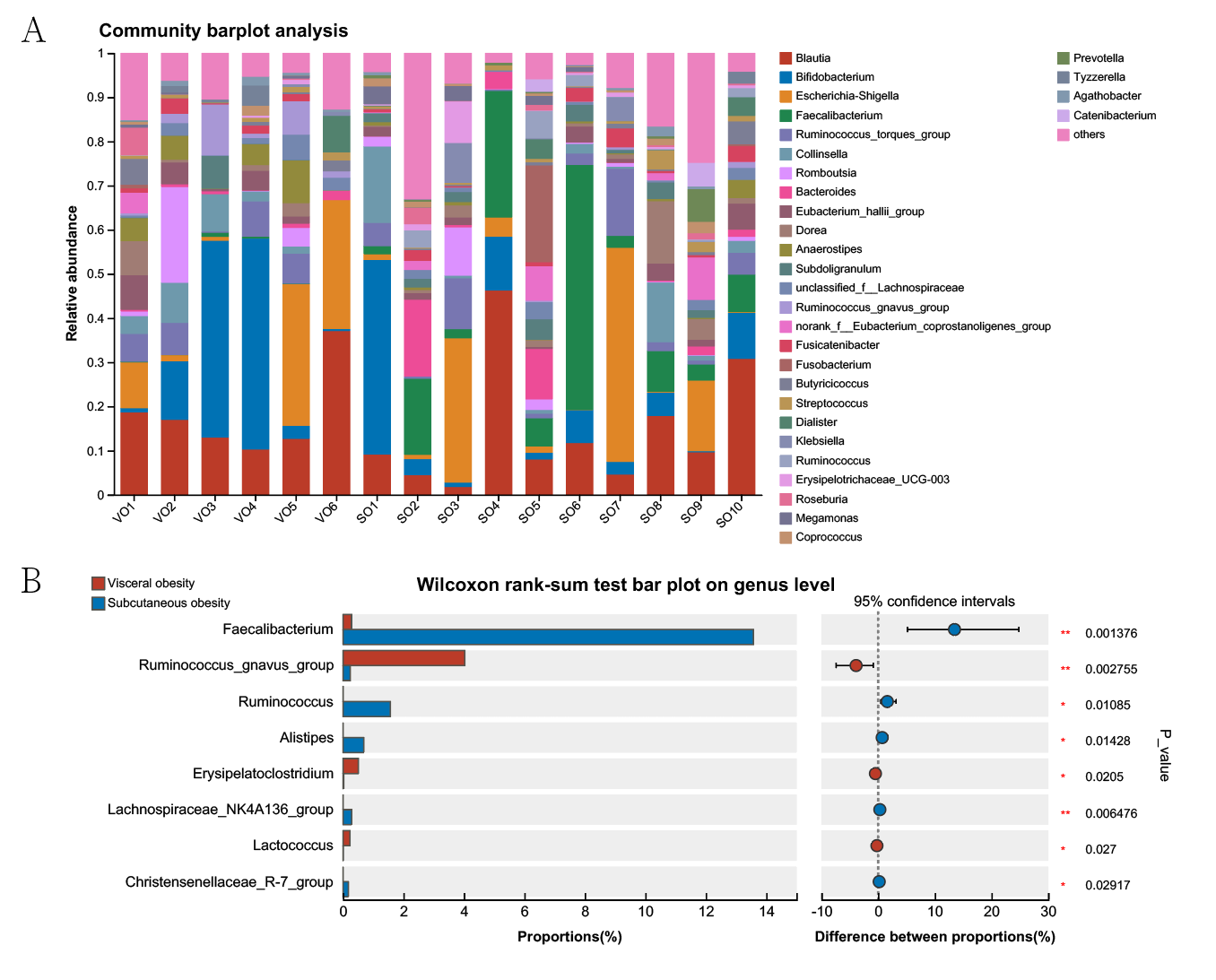


**Supplementary Figure 10.** **Gut microbiota composition and differences between visceral and subcutaneous obesity in the validation cohort.**

A. Community barplot analysis shows the relative abundance of gut microbiota in patients with visceral obesity (VO) and subcutaneous obesity (SO). Different colors represent different bacterial genera, and their relative abundance is visualized as stacked bar plots across samples.

B. Wilcoxon rank-sum test bar plot at the genus level compares the differences in gut microbial genera between visceral obesity (red) and subcutaneous obesity (blue) groups. The x-axis shows the proportion of each genus, and the dot plot on the right illustrates the difference between the two groups with 95% confidence intervals. Significant differences (*P* < 0.05) are marked with asterisks, with genera like *Faecalibacterium* and *Ruminococcus_gnavus_group* showing notable differences in abundance between the two obesity types.

**Supplementary Table 1. Baseline characteristics of participants in the validation cohort.**

| **Characteristics** | Visceral obesity | Subcutaneous obesity |  |
| --- | --- | --- | --- |
|  | (n=6) | (n=10) | *P*-values |
| VFV | 926.50 (195.46) | 534.80 (247.12) | 0.005 |
| SFV | 962.83 (173.33) | 1468.00 (622.04) | 0.075 |
| VSR | 0.96 (0.06) | 0.37 (0.10) | <0.001 |
| Gender, male, % | 1 (16.67) | 0 (0.00) | 0.79 |
| Age | 45.67 (10.97) | 27.80 (4.71) | <0.001 |
| BMI | 35.37 (4.84) | 35.45 (7.24) | 0.98 |
| HbAlc% | 6.47 (1.11) | 5.52 (0.66) | 0.049 |
| ALT | 66.08 (78.74) | 22.81 (5.11) | 0.098 |
| AST | 112.95 (111.46) | 30.28 (15.90) | 0.033 |
| HDL-C | 1.25 (0.16) | 1.20 (0.20) | 0.61 |
| TG | 2.26 (1.33) | 1.55 (0.45) | 0.139 |
| LDL-C | 3.38 (1.03) | 3.09 (0.90) | 0.561 |
| Glucose | 6.09 (1.20) | 4.95 (0.90) | 0.047 |
| Total Cholesterol | 5.20 (1.24) | 5.03 (1.37) | 0.802 |
| C-Peptide | 1.49 (0.48) | 1.60 (0.66) | 0.718 |
| Insulin | 27.10 (15.90) | 36.31 (20.79) | 0.368 |

**Abbreviations**: VFV: Visceral Fat Volume; SFV: Subcutaneous Fat Volume; VSR: Visceral-to-Subcutaneous Fat Ratio; HbAlc: Hemoglobin A1c; GGT: Gamma-Glutamyl Transferase; ALT: Alanine Aminotransferase; AST: Aspartate Aminotransferase; HDL-C: High-Density Lipoprotein Cholesterol; TG: Triglycerides; LDL-C: Low-Density Lipoprotein Cholesterol.

*P* < 0.05 was considered statistically significant.
